# SINCERITIES: inferring gene regulatory networks from time-stamped single cell transcriptional expression profiles

**DOI:** 10.1093/bioinformatics/btx575

**Published:** 2017-09-14

**Authors:** Nan Papili Gao, S M Minhaz Ud-Dean, Olivier Gandrillon, Rudiyanto Gunawan

**Affiliations:** 1 Institute for Chemical and Bioengineering, ETH Zurich, Zurich, Switzerland; 2 Swiss Institute of Bioinformatics, Lausanne, Switzerland; 3 Department of Environmental Health Sciences, Mailman School of Public Health, Columbia University, New York, NY, USA; 4 Laboratory of Biology and Modelling of the Cell, Univ Lyon, ENS de Lyon, Univ Claude Bernard, CNRS UMR, INSERM Lyon, France; 5 Inria Team Dracula, Inria Center Grenoble Rhône-Alpes, Rhône-Alpes, France

## Abstract

**Motivation:**

Single cell transcriptional profiling opens up a new avenue in studying the functional role of cell-to-cell variability in physiological processes. The analysis of single cell expression profiles creates new challenges due to the distributive nature of the data and the stochastic dynamics of gene transcription process. The reconstruction of gene regulatory networks (GRNs) using single cell transcriptional profiles is particularly challenging, especially when directed gene-gene relationships are desired.

**Results:**

We developed SINCERITIES (SINgle CEll Regularized Inference using TIme-stamped Expression profileS) for the inference of GRNs from single cell transcriptional profiles. We focused on time-stamped cross-sectional expression data, commonly generated from transcriptional profiling of single cells collected at multiple time points after cell stimulation. SINCERITIES recovers directed regulatory relationships among genes by employing regularized linear regression (ridge regression), using temporal changes in the distributions of gene expressions. Meanwhile, the modes of the gene regulations (activation and repression) come from partial correlation analyses between pairs of genes. We demonstrated the efficacy of SINCERITIES in inferring GRNs using *in silico* time-stamped single cell expression data and single cell transcriptional profiles of THP-1 monocytic human leukemia cells. The case studies showed that SINCERITIES could provide accurate GRN predictions, significantly better than other GRN inference algorithms such as TSNI, GENIE3 and JUMP3. Moreover, SINCERITIES has a low computational complexity and is amenable to problems of extremely large dimensionality. Finally, an application of SINCERITIES to single cell expression data of T2EC chicken erythrocytes pointed to BATF as a candidate novel regulator of erythroid development.

**Availability and implementation:**

MATLAB and R version of SINCERITIES are freely available from the following websites: http://www.cabsel.ethz.ch/tools/sincerities.html and https://github.com/CABSEL/SINCERITIES. The single cell THP-1 and T2EC transcriptional profiles are available from the original publications (Kouno *et al.*, 2013; Richard *et al.*, 2016). The *in silico* single cell data are available on SINCERITIES websites.

**Supplementary information:**

Supplementary data are available at *Bioinformatics* online.

## 1 Introduction

Cell profiling technologies have enabled scientists to measure intracellular molecules (DNA, RNA, proteins, metabolites) at whole-genome level and down to single cell resolution. Over the last decade, high-throughput single cell assays have experienced tremendous progress, thanks to advanced microfluidics techniques and increased sensitivity in cell profiling assays. For example, the Fluidigm Dynamic Array platform employs integrated fluidics circuitry to capture single cells (up to 96 cells per run) for transcriptional expression profiling using quantitative RT-PCR (qRT-PCR) or RNA-sequencing (RNA-seq) (Pieprzyk and High, 2009). Furthermore, the arrival of barcoding strategies will bring such approaches to unprecedented resolution (Rosenberg *et al.*, 2017). The ability to assay individual cells and to examine intra-population cellular heterogeneity brings great benefits to fields such as stem cell and cancer biology. In the last few years, single cell analyses have demonstrated the ubiquity of cellular heterogeneity, even within cell populations or cell types that have been traditionally perceived as homogeneous (Buettner *et al.*, 2015; Gupta *et al.*, 2011; Kumar *et al.*, 2014; Pollen *et al.*, 2014; Shalek *et al.*, 2014). Meanwhile, many single cell studies have provided evidence for the physiological roles of cell-to-cell variability in normal and diseased cells (Chang *et al.*, 2008; Fang *et al.*, 2013; Lee *et al.*, 2014; Kim *et al.*, 2015; Richard *et al.*, 2016).

Single cell transcriptional profiling overcomes many issues associated with population-average or bulk data that mask cellular heterogeneity [e.g. Simpson’s paradox (Simpson, 1951)], thereby presenting new means for understanding biology. Bioinformatics tools for analyzing single cell expression data have proliferated in recent years (Bacher *et al.*, 2016; Liu and Trapnell, 2016; Stegle *et al.*, 2015). A class of these algorithms concerns with the deconvolution of cell populations and tissues to elucidate population substructures and identify known and novel cell subtypes (Amir *et al.*, 2013; Buettner *et al.*, 2014; Haghverdi *et al.*, 2015; Pierson *et al.*, 2015; Xu and Su, 2015). These algorithms often apply or modify existing clustering and dimensionality reduction algorithms, such as PCA, tSNE and diffusion maps, to accommodate single cell data. Another class of algorithms deals with the ordering of cells within the cell population along a perceived unique transition path between different cell states [e.g. Monocle (Trapnell *et al.*, 2014), Wanderlust (Bendall *et al.*, 2014), SCUBA (Marco *et al.*, 2014) and TSCAN (Ji and Ji, 2016)]. Such cell ordering produces a trajectory in the state space of gene expression corresponding to a physiological transition, such as stem cell differentiation process.

The third class of algorithms considers gene regulatory network (GRN) inference. A GRN is a network graph, where the nodes of this graph represent genes and the edges represent gene-gene interactions. The most common gene networks created from single cell transcriptional data have undirected edges [see for example (Kouno *et al.*, 2013; Pina *et al.*, 2015; Richard *et al.*, 2016)], where such edges indicate associations among genes, for example co-expression or co-regulation relationships. In contrast, the focus of our work is inferring GRNs with directed edges, where an edge pointing from gene *i* to gene *j* implies that the protein product(s) of gene *i* directly or indirectly regulates the expression of gene *j* (e.g. gene *i* encodes a transcription factor of gene *j*). The edges may also have signs, representing the modes of the gene regulation: positive for activation and negative for repression. In comparison to the other two classes of algorithms, there have been lesser algorithmic developments on the inference of such GRNs from single cell transcriptional profiles, possibly because of the extreme difficulty in this task (Bacher *et al.*, 2016; Liu and Trapnell, 2016; Stegle *et al.*, 2015).

One of the challenges in using single cell expression data for GRN inference is the zero-inflated characteristic of the dataset, resulting from both technical dropouts (mainly in RNA-seq data) and the stochastic bursty dynamics of the gene expression process (Bacher *et al.*, 2016; Coulon *et al.*, 2010; Liu and Trapnell, 2016; Stegle *et al.*, 2015). In addition, single cell profiling techniques such as qRT-PCR and RNA-seq use cell lysates. Consequently, the resulting data provide only cross-sectional information of the cell population. A few GRN inference methods have previously been proposed based on Boolean network model (Chen *et al.*, 2015; Lim *et al.*, 2016; Moignard *et al.*, 2015), stochastic gene expression model (Teles *et al.*, 2013) and a combination of machine learning and nonlinear differential equation model (Matsumoto *et al.*, 2017; Ocone *et al.*, 2015). However, none of these methods use time point information of the cells directly in the GRN inference. In general, temporal data possess more information than static or single time-point data, especially for the determination of causal networks (Bar-Joseph *et al.*, 2012). For these reasons, here we consider time-stamped cross-sectional single cell transcriptional profiles, i.e. the expression profiles of single cells taken at multiple time points after cell stimulation. Such type of dataset is commonly generated in studies of cell differentiation process, where cells are induced to differentiate at the beginning of the experiment and are then collected at multiple time points for single cell analysis (Chu *et al.*, 2016; Kouno *et al.*, 2013; Richard *et al.*, 2016).

In this work, we created a network inference algorithm, called SINCERITIES (SINgle CEll Regularized Inference using TIme-stamped Expression profileS). The GRN inference was formulated as regularized linear regressions based on temporal changes of the gene expression distributions. The modes of the gene regulations, i.e. the signs of the edges, were determined using partial correlation analyses. We demonstrated the efficacy of SINCERITIES using *in silico* time-stamped single cell expression profiles, as well as time-stamped cross-sectional transcriptional profiles of THP-1 human myeloid monocytic leukemia cells (Kouno *et al.*, 2013) and T2EC chicken erythrocytes (Richard *et al.*, 2016). We also compared SINCERITIES to existing GRN inference algorithms developed for time series expression data, namely TSNI (Bansal *et al.*, 2006) and JUMP3 (Huynh-Thu and Sanguinetti, 2015), and to a tree-based GRN inference algorithm GENIE3 (Huynh-Thu *et al.*, 2010). The case studies illustrated the efficacy of SINCERITIES in extracting accurate GRN, by taking advantage of temporal information in time-stamped single cell expression data.

## 2 Materials and methods

### 2.1 Gene regulatory network inference using SINCERITIES

**Fig. 1.**
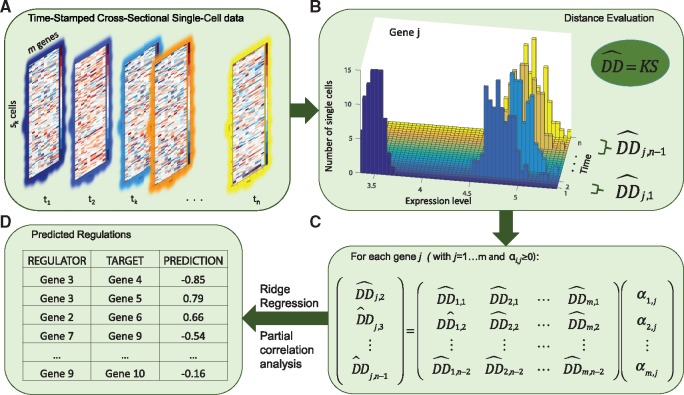
The workflow of SINCERITIES. (**A**) Input: time-stamped cross-sectional data of gene expression. (**B**) Step 1: calculation of normalized distribution distance of gene expression distributions over each time step; (**C**) Step 2: formulation of the GRN inference as a linear regression problem; (**D**) Output: edge predictions of the GRN

Figure 1 illustrates the main steps of the gene regulatory network inference in SINCERITIES. In the following, let *m* be the number of genes, *n* be the number of measurement time points, and *s_k_* be the number of cells in the *k*th time point sample (*k = *1, 2, …, *n*). The time-stamped cross-sectional dataset (see Fig. 1A) comprises *n* data matrices $E_{s_{k}\times m}$, where the matrix element $E_{i_{k},j}$ is the transcriptional expression value of gene *j*, i.e. the amount of mRNA molecules of gene *j* in the *i*th cell at the *k*th time point. SINCERITIES is based on the assumption that changes in the expression of a transcription factor (TF) will alter the expression of the target genes. Thus, in the first step of SINCERITIES (see Fig. 1B), we quantify the temporal changes in the expression of each individual gene by computing the distance of the marginal gene expression distributions between two subsequent time points. While the most obvious distributional distance (DD) metric is the mean difference, the transcriptional regulation of a gene could alter the gene expression distribution beyond its first moment (Altschuler and Wu, 2010; Vallejos *et al.*, 2016). In SINCERITIES, we make use of the information contained in the single cell gene expression dataset, particularly changes in the gene expression distributions, for the purpose of GRN inference. In the current implementation of SINCERITIES, we have chosen the Kolmogorov–Smirnov (KS) distance, i.e. the maximum absolute difference between two cumulative density functions, as the DD metric (Massey, 1951). However, if desired and whenever appropriate, other DD metrics, such as the mean difference, Anderson-Darling (AD) statistics (Anderson and Darling, 1952) and the Cramér–von Mises (CM) criterion (Anderson, 1962), could also be used in place of the KS distance (see also Section 2.2).

In order to establish directed edges in the GRN, we adopted the Granger causality concept (Granger, 1969), where the direction of an edge indicates predictive causality, i.e. past data have the information for predicting the future observations. More specifically, in SINCERITIES, we formulated the GRN inference problem, in which the changes in the expression of TFs in a given time window are used to ‘predict’ the shifts in the gene expression distributions of the corresponding target genes in the next time window. Since the time windows may not necessarily be uniform, the DD values are normalized by the time step size. As shown in Figure 1C, the GRN inference in SINCERITIES involves solving *m* independent linear regressions. More specifically, for each gene *j*, we formulate a linear regression using the normalized DDs of this gene at time windows *l + *1, denoted by ${\hat{DD}}_{j,l+1}$ (l=1, 2,…,n-2), as the response (dependent) variable, while setting the normalized DDs of all other genes from the previous time window l (${\hat{DD}}_{p,l},\;p=1,\;2,\ldots ,m$) as the regressor (independent) variables. The linear regression is thus given by:

(1) $${\hat{DD}}_{j,l+1}={\alpha_{1,j}\hat{DD}}_{1,l}+{\alpha_{2,j}\hat{DD}}_{2,l}+\cdots +{\alpha_{m,j}\hat{DD}}_{m,l}$$

where *α_p, j_* is the regression coefficient describing the influence of gene *p* on gene *j*. The least square solution vector $\alpha_{j}^{*}$ is constrained to be non-negative since the normalized DDs take only non-negative values. In formulating the regression problem above, we have followed the standard mathematical statement of the Granger causality, and therefore made a simplification in which the relationship between the DDs of the regulators and those of the target gene is linear. While higher order (nonlinear) relationships could be incorporated into the regression problem above, the applications of SINCERITIES to *in silico* and actual single cell expression dataset below demonstrated that the linear approximation could provide reasonably accurate predictions of the GRN structure.

The linear regression above is often underdetermined as the number of genes typically exceeds the number of time windows. For this reason, we employ a penalized least square approach to obtain $\alpha_{j}^{*}$ using an L_2_-norm penalty, also known as ridge regression or Tikhonov regularization (see Section 2.3 for more details). SINCERITIES relies on GLMNET (Friedman *et al.*, 2010) to compute the solution vector $\alpha_{j}^{*}\;$for each gene *j*, using leave-one-out cross-validation (LOOCV) for determining the weight of the penalty term. Upon completion, SINCERITIES produces a ranked list of all possible edges in the GRN (a total of *m*^2^ edges) in descending order of *α_p, j_* values (see Fig. 1D). A larger *α_p, j_* indicates higher confidence that the corresponding edge exists (i.e. the edge *p → j*). For the mode (sign) of the gene regulatory edges, SINCERITIES uses partial correlation analyses on the expressions of every gene pair, controlling for the other genes (see Section 2.4). The sign of an edge is set to the sign of the corresponding partial correlation. In other words, a positive (negative) correlation is taken as an indication of activation (repression).

Presently, SINCERITIES cannot directly handle single cell data from stem cell differentiation process that produces more than one cell type (i.e. branching). In such a scenario, a pre-processing step is needed to group cells into individual cell lineages [for example, using time-variant clustering (Huang *et al.*, 2014)], and SINCERITIES could subsequently be applied to data from each differentiation branch. In the case studies, we tested SINCERITIES performance in inferring moderately sized GRNs. While there exist no technical limitation in applying SINCERITIES to single cell expression data with many more genes, for example using RNA-seq data, we expect that network inferability would become the limiting issue in such an inference (Szederkényi *et al.*, 2011; Ud-Dean and Gunawan, 2014). Finally, the current implementation of LOOCV in SINCERITIES requires at least five time points. With n=5, the regression in Eq. (1) comprises n-2 = 3 equations, which is the minimum number of samples in the LOOCV for computing the average and standard deviation of the test errors.

### 2.2 Distribution distance

In SINCERITIES, we used the Kolmogorov–Smirnov distance to quantify the distance between two cumulative distribution functions of gene expressions from subsequent time points, according to

(2) $${DD}_{j,l}=\max\left|F_{t_{l+1}}\left(E_{j}\right)-F_{t_{l}}\left(E_{j}\right)\right|$$

where *DD_j, l_* denotes the distributional distance of gene *j* expression *E_j_* between time points *t_l_* and *t_l+_*_1_ (*l = *1, 2, …, *n *-* *1) and $F_{t_{l}}\left(E_{j}\right)$ denotes the cumulative distribution function of *E_j_* at time *t_l_*. We also evaluated three additional DD metrics, namely the mean difference, AD statistics and CM criterion (Anderson, 1962) (see Supplementary Material and Supplementary Table S1). As shown in the case study using *in silico* single cell data, the performance of SINCERITIES did not depend sensitively on the DD metrics used. In order to accommodate non-uniformity in the sampling times, we normalized *DD_j, l_* with respect to the time window size, as follows:

(3) $${\hat{DD}}_{j,l}=\frac{{DD}_{j,l}}{\Delta t_{l}}$$

where ${\hat{DD}}_{j,l}$ denotes the normalized distribution distance of gene *j* in the time window between *t_l_* and *t_l+_*_1_ with $\Delta t_{l}=t_{l+1}-t_{l}$.

### 2.3 Ridge regression

As shown in Figure 1C and Eq. (1), for each gene *j*, we solved a linear regression problem of the form: y=Xα, where **y** denotes the *n-*2 vector of $\hat{DD}$ distances of gene *j* corresponding to time windows Δ*t*_2_ to Δ*t_n-_*_1_, and **X** denotes the (*n-*2)×*m* matrix of $\hat{DD}$ distances corresponding to time windows Δ*t*_1_ to Δ*t_n-_*_2_, for all genes. To obtain the solution vector **α**, we performed a ridge regression penalized least square optimization as follows:

(4) $$\min_{\alpha }{\left\|\left\|y-X\alpha \right\|\right\|}_{2}^{2}+\frac{1}{2}\lambda {\left\|\left\|\alpha \right\|\right\|}_{2}^{2}$$

with the constraint that $\alpha_{i}\geq 0$. We used GLMNET algorithm (MATLAB) to generate the regularization path, i.e. the solution α as a function of different λ values (Friedman *et al.*, 2010). In addition to ridge regression, we also tested SINCERITIES with two other penalty functions: the ‘Least Absolute Shrinkage and Selection Operator’ (Lasso) L_1_-norm penalty (Tibshirani, 1996) and the elastic-net penalty (Zou and Hastie, 2005). These alternative penalty functions however led to less accurate GRN predictions than the ridge regression (for further details, see Supplementary Material and Supplementary Table S2).

The optimal weight factor *λ* above is typically data dependent. Here, we performed a leave-one-out cross validation (Kohavi, 1995) to determine the optimal weight factor *λ.* In LOOCV, we allocated one row of **y** and **X** as the test dataset and the remaining as the training dataset. Then, we generated the regularization path for the training dataset using GLMNET, and computed the error of predicting the test dataset as a function of *λ.* We repeated this exercise for every permutation of test and training dataset assignment, and selected the optimal *λ* that minimized the average prediction error. Finally, we ran GLMNET on the full dataset and took the solution α* that corresponded to the optimal *λ* value above.

### 2.4 Partial correlation analysis

In order to determine the mode (sign) of gene regulatory relationships, we performed the Spearman rank partial correlation analysis. More specifically, for every pair of genes, we calculated the Spearman rank partial correlation coefficient of the combined expressions from all time points, while controlling for the other genes. The sign of the regulatory edge pointing from gene *i* to gene *j* was set equal to the sign of the partial correlation coefficient. Note that by using correlation, the sign of the edge pointing from gene *i* to gene *j* is equal to the sign of the edge pointing from gene *j* to gene *i.*

### 2.5 *In silico* data generation

For testing the performance of SINCERITIES, we used GeneNetWeaver (GNW) to randomly generate 10-gene and 20-gene random subnetworks of *Escherichia coli* and *Saccharomyces cerevisiae* (yeast) GRNs. After removing self-regulations, we simulated *in silico* single cell expression data using the following stochastic differential equation (SDE) model of the mRNA (Pinna *et al.*, 2010):

(5) $${dx}_{j}(t)=V\left(\beta \prod_{i=1}^{n}\left(1+A_{i,j}\frac{x_{i}\left(t\right)}{x_{i}\left(t\right)+1}\right)-\theta x_{i}\left(t\right)\right)dt+\sigma x_{j}(t)dW(t)$$

where *x_j_* describes the mRNA level of gene *j*, *A_i, j_* denotes the regulation of the expression of gene *j* by gene *i, β* denotes the basal transcriptional rate, *θ* denotes the mRNA degradation rate constant, and *σ* and *V* are scaling parameters. The term *dW(t)* denotes the random Wiener process, simulating the intrinsic stochastic dynamics of gene expression (Wilkinson, 2009). We set *A_ij_* to 1 for gene activation, −1 for gene repression, and 0 otherwise. For the main dataset in the case study, we set the parameters to the following: *V *=* *30, *β = *1, *θ = *0.2 and *σ = *0.1.

We simulated the SDE model above using the Euler-Maruyama method (Higham, 2001) with an initial condition *x_j_*(0) set to 0 for every gene, until the gene expression reached steady state (*t *= 3 arbitrary time unit). For each GRN structure, we generated 100 stochastic trajectories for each time point (a total of 8 × 100 = 800 independent trajectories for 8 time points), representing 100 single cells. The simulations above mimicked the scenario where single cells are lysed for gene expression profiling. To test the robustness of SINCERITIES with respect to the intrinsic noise in gene expression and to the number of sampling time points, we further generated two additional datasets from the 10-gene *E.coli* and yeast gold standard GRNs, by varying *σ* parameter between 0.1 and 0.4 with a step of 0.1 (see Table 1A) and by selecting the first *n* time points from the following set *t* = 0.51, 0.60, 0.74, 1.2, 1.3, 1.5, 1.8, 2.2, 2.6, 3, where *n* is between 6 and 10. The time points were selected to exclude the time period during which the mRNA level rose quickly from the initial concentration. This initial increase was a consequence of starting the simulations from *x_j_*(0) = 0, and did not necessarily reflect the gene regulatory actions.

**Table 1.** Robustness of SINCERITIES to (A) intrinsic stochastic noise and (B) number of time points

10-GENE NETWORK		
	AUROC	AUPR
σ	A	
0.1	0.78 ± 0.11	0.34 ± 0.17
0.2	0.76 ± 0.10	0.33 ± 0.16
0.3	0.66 ± 0.10	0.22 ± 0.10
0.4	0.60 ± 0.10	0.17 ± 0.07
Time points	B	
10	0.78 ± 0.16	0.32 ± 0.17
9	0.79 ± 0.11	0.39 ± 0.22
8	0.78 ± 0.11	0.34 ± 0.17
7	0.80 ± 0.10	0.36 ± 0.20
6	0.78 ± 0.11	0.37 ± 0.20

## 3 Results

### 3.1 Evaluation of SINCERITIES using *in silico* data

To evaluate the efficacy of SINCERITIES, we simulated *in silico* time-stamped single cell expression datasets using 10-gene and 20-gene gold standard GRNs. The gold standard GRNs comprised 40 random subnetworks of *E.coli* and *S.cerevisiae* GRNs, i.e. ten networks for each size and from each species (see Supplementary File), generated using GeneNetWeaver (Schaffter *et al.*, 2011). For the main dataset, we simulated single cell gene expression data for 100 cells at 8 unevenly spaced time points using a stochastic differential equation model (see Section 2.5). In order to test the robustness of SINCERITIES with respect to the number of sampling time points and to the degree of stochasticity in the gene expression, we further generated additional datasets using the 10-gene GRNs above, for varying degrees of intrinsic noise (by changing *σ* parameter) and different numbers of sampling time points (see Section 2.5). In the gold standard GRNs, we assumed that there exist no self-regulatory edges, since some of the existing algorithms used in the comparison, namely GENIE3 and JUMP3, could not identify such edges.

We assessed the performance of SINCERITIES by evaluating the areas under the Receiver Operating Characteristic (AUROC) and the Precision-Recall curve (AUPR). Higher AUROC and AUPR values indicate more accurate GRN predictions. For this purpose, we computed the numbers of true positive (TP), true negative (TN), false positive (FP) and false negative (FN) edges by comparing the regulatory edges in the gold standard network with the top *q* edges from the ranked list output of SINCERITIES. When considering GRNs with signed edges, a true positive prediction referred to the correct prediction of an edge and its sign. The ROC curve was constructed by plotting the true positive rates (TPR = TP/(TP + FN)) versus the false positive rates (FPR = FP/(FP + TN)) for increasing *q* ($q=1,\;2,\ldots ,m^{2}$). Similarly, the precision (TP/(TP + FP)) versus recall (TP/(TP + FN)) curve was plotted for increasing *q*.

**Fig. 2.**
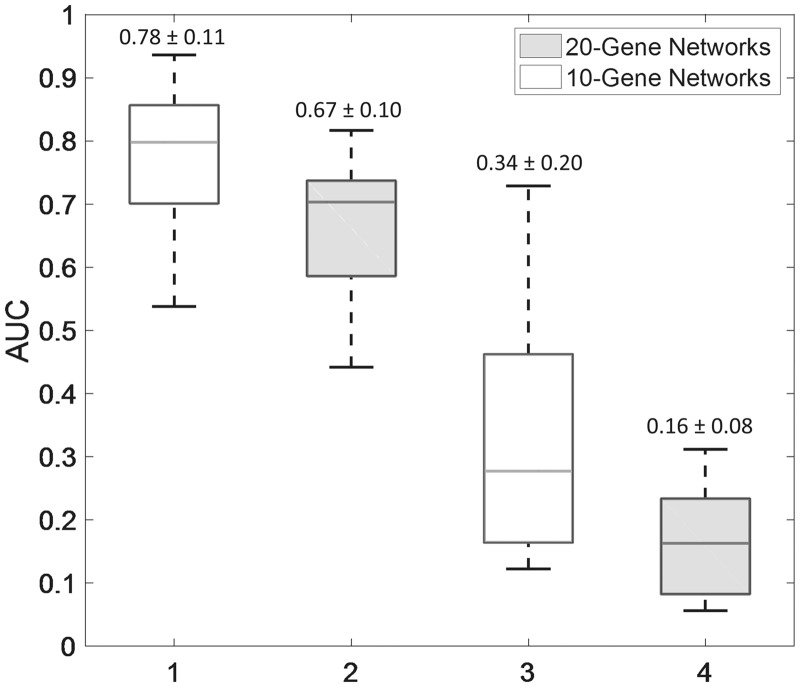
Performance of SINCERITIES in inferring gold standard GRNs. The AUROC and AUPR values are given in Supplementary Table S1

Figure 2 shows the AUROC and AUPR values of SINCERITIES predictions for the main dataset, respecting the signs of the gene regulatory edges. As expected, the larger GRNs (20-gene) were more difficult to infer than the smaller GRNs (10-gene), as indicated by the lower AUROC and AUPR values. GRNs with a larger mean or maximum distance among the genes (nodes) were also more difficult to infer (see Supplementary Fig. S6). As we have shown previously (Ud-Dean and Gunawan, 2014), an indirect regulation of a gene by another (i.e. a network distance of 2 or higher) is often predicted as a direct regulation, leading to a false positive error. Meanwhile, Table 1 gives the mean AUROC and AUPR values of SINCERITIES for the additional single cell dataset. In general, the performance of SINCERITIES decreased slightly with increasing intrinsic stochasticity. On the other hand, decreasing the number of time points did not appreciably change the performance of SINCERITIES.

We further compared the performance of SINCERITIES to three other network inference methods, namely TSNI (Bansal *et al.*, 2006), GENIE3 (Huynh-Thu *et al.*, 2010) and JUMP3 (Huynh-Thu and Sanguinetti, 2015). TSNI (Time Series Network Inference) is a GRN inference algorithm developed for time series gene expression data, relying on a linear ordinary differential equation model of the gene transcriptional process (Bansal *et al.*, 2006). Meanwhile, GENIE3 (GEne Network Inference with Ensemble of trees) employs on a tree-based ensemble strategy using either random forest or extra-trees algorithms (Huynh-Thu *et al.*, 2010). GENIE3 was among the top performers in DREAM 4 and DREAM 5 network inference challenges (Marbach *et al.*, 2009, 2012). Recently, GENIE3 has also been applied to single cell data as a preliminary step to obtain the skeleton of the GRN (Ocone *et al.*, 2015). Lastly, JUMP3 uses a hybrid strategy combining non-parametric decision trees approach with dynamical ON/OFF modelling, to infer GRNs from time series expression data (Huynh-Thu and Sanguinetti, 2015). Since TSNI and JUMP3 require time series (longitudinal) data, we applied these methods to the (population) averages of the single cell gene expression data from each time point. Among the three previous methods, only TSNI generates GRN predictions with signed edges.

**Fig. 3.**
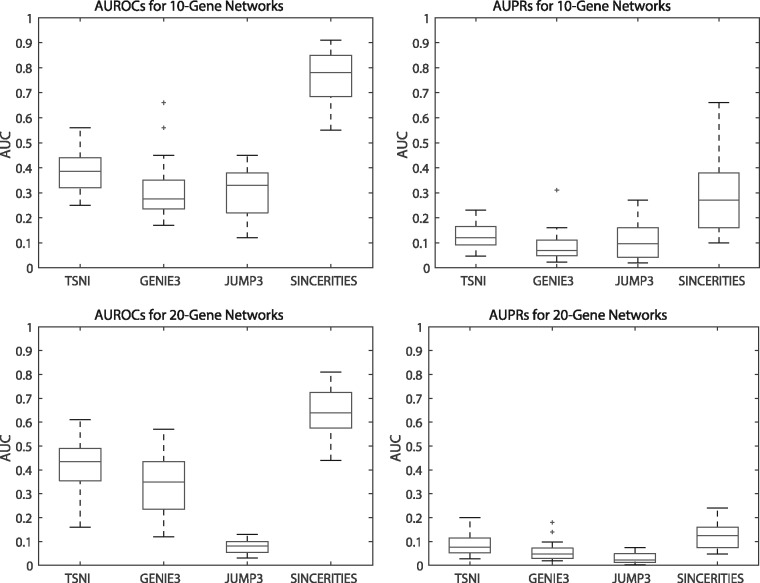
Performance comparison among TSNI, GENIE3, JUMP3 and SINCERITIES. (**A**) AUROC and (**B**) AUPR values for 10-gene gold standard GRNs. (**C**) AUROC and (**D**) AUPR values for 20-gene gold standard GRNs. The AUROC and AUPR values are given in Supplementary Table S3

Figure 3 compares the AUROC and AUPR values of SINCERITIES and the three other methods mentioned above. The AUROC and AUPR values for TSNI and SINCERITIES were computed by respecting for the signs of the edges. However, for unsigned GRN predictions from GENIE3 and JUMP3, the AUROC and AUPR values were based only on the existence of the regulatory edges (ignoring signs). The results showed that SINCERITIES significantly outperformed all of these methods (*P*-value < 0.05, paired t-tests) (see Supplementary Table S3).

### 3.2 Inferring GRN driving THP-1 differentiation

In the following, we applied SINCERITIES to infer the GRN that drives the differentiation of monocytic THP-1 human myeloid leukemia cell differentiation into macrophages. The time-stamped cross-sectional single cell data came from qRT-PCR expression profiling of 45 TFs in 960 THP-1 cells that were collected at 8 distinct time points (0, 1, 6, 12, 24, 48, 72, 96 h) after stimulation by 12-myristate 13-acetate (PMA) (Kouno *et al.*, 2013). This dataset provided a good benchmark inference problem as the GRN of THP-1 differentiation has previously been constructed using deep sequencing (deepCAGE) and RNA interference (RNAi) experiments (Tomaru *et al.*, 2009; Vitezic *et al.*, 2010). More specifically, we used a previously constructed anti-/pro-differentiation TF network (Tomaru *et al.*, 2009) as the gold standard network for evaluating the performance of SINCERITIES and the three existing inference methods above.

We applied SINCERITIES as well as TSNI, GENIE3 and JUMP3 to reconstruct the GRN of THP-1 differentiation using the single cell expression data above. The AUROC and AUPR values were evaluated against the gold standard network. We noted that only 20 TFs in the RNAi study overlapped with the set of genes in the single cell study (Kouno *et al.*, 2013). Therefore, while the GRN inferences were done for 45 TFs, the calculation of AUROCs and AUPRs was based on the regulatory edges among the common set of 20 TFs. Again, for GENIE3 and JUMP3, the AUROC and AUPR values did not take into account the modes (signs) of the regulatory edges.

Table 2 gives the AUROCs and AUPRs for the four network inference strategies. For SINCERITIES, we reported the AUROC and AUPR values both with and without the mode (signs) of the gene regulations. The AUROC and AUPR values of SINCERITIES for the unsigned GRN prediction were similar to those using *in silico* data. As expected, the AUROC and AUPR values for the signed GRN prediction from SINCERITIES was lower, but only slightly. TSNI, GENIE3 and JUMP3 performed worse than SINCERITIES, and often did not give much better predictions than a random network (AUROC = 0.50).

**Table 2.** Performance comparison among TSNI, GENIE3, JUMP3 and SINCERITIES in inferring the GRN of THP-1 cell differentiation

	AUROC	AUPR
TSNI	0.44	0.11
GENIE3	0.46	0.23
JUMP3	0.52	0.16
SINCERITIES (without sign)	0.70	0.33
SINCERITIES (with sign)	0.64	0.25

### 3.3 Inferring novel regulator(s) of T2EC differentiation

In this application, we used SINCERITIES to infer the GRN associated with the differentiation process of T2EC chicken erythrocytic cells. The single cell RT-qPCR dataset comprised 90 genes at 0, 8, 24, 33, 48 and 72 h after induction to differentiate (Richard *et al.*, 2016). The 90 genes were selected based on differential expression and clustering analysis of time-series bulk RNA-seq data from induced and uninduced T2EC cells, and included highly significantly upregulated and downregulated genes as well as non-differentially regulated genes. Here, there exists no gold standard network against which we could compare the accuracy of the inferred GRN. The goal of the analysis was to identify candidate novel genes that drive the differentiation process. For this purpose, we employed the inferred GRN from SINCERITIES, accounting for any edges with non-zero α coefficients, and ordered the genes based of the decreasing ratio between the out- and in-degree (Kouno *et al.*, 2013). More specifically, for each gene *j*, we computed the out- and in-degree as the number of (target) genes that are regulated by gene *j* and the number of (regulator) genes those that regulate gene *j*, respectively. In the following, we divided the ordered gene list into three roughly equisized groups: upstream genes (out/in-degree ≥ 5.5), midstream genes (5 > out/in-degree ≥ 1.1) and downstream genes (out/in-degree < 1.1) (see Supplementary Table S4).

Table 3 shows the enriched gene ontology (GO) terms for the up-, mid- and downstream genes [using TOPPCLUSTER (Kaimal *et al.*, 2010)]. We found statistically significant enrichments (Bonferroni-corrected *P*-value < 0.05) only for the upstream and midstream genes. Among the enriched GO terms in the upstream gene list, the sterol and cholesterol biosynthesis have previously been implicated in the differentiation of T2EC cells (Richard *et al.*, 2016). In addition, the cell activation process was significantly enriched among the upstream genes, while the ERBB2 signaling pathway was enriched among the midstream genes. A repeat of the GO enrichment analysis using the top 500 edges from SINCERITIES produced a similar outcome with sterol and cholesterol biosynthesis and cell activation being the enriched GO terms among the up- and mid-stream genes (see Supplementary Table S5). In this case, ERBB2 signaling was not significantly enriched. Below, we thus focused on the cell activation process.

**Table 3.** Gene Ontology Enrichment Analysis of Up-, Mid- and downstream genes in T2EC differentiation

Enriched GO Biological Process Terms	−log10(*P*)		
	Upstream	Midstream	Downstream
Cholesterol biosynthetic process	5.8428*	2.5237	2.5832
Secondary alcohol biosynthetic process	5.8428*	2.5237	2.5832
Sterol biosynthetic process	5.6780*	2.4438	2.5032
Cell activation	4.6132*	1.6896	0.3495
ERBB2 signaling pathway	1.2045	4.4906*	–

(*) Bonferroni-corrected *P*-value < 0.05.

The genes in the T2EC dataset related to the cell activation comprise BATF, BCL11A, BPI, CD44, EGFR, LCP1, PIK3CG, PTPRC and SNX27. A subset of the genes above has known roles in erythroid development. Particularly, BCL11A is a TF that regulates globin gene expression (Sankaran *et al.*, 2009). CD44 is expressed in erythrocytes, and participates in the cell adhesion function (Telen, 2000). Furthermore, EGFR (Gandrillon *et al.*, 1999) and PIK3CG (Dazy *et al.*, 2003) have been previously shown to promote self-renewal state and to inhibit cell differentiation in T2EC. In agreement with the previous observation, the expression of EGFR was downregulated during T2EC differentiation process (see Supplementary Fig. S5).

The remaining genes however have no reported roles in erythroid differentiation. The possible involvements of BATF, BPI and LCP1 in T2EC differentiation have also been raised in the original analysis of the dataset (see Supplementary Fig. S8 in Richard *et al.*, 2016). A TF enrichment analysis of the cell activation gene set above using Enrichr (ENCODE TF ChIP-seq) (Chen *et al.*, 2013) indicated SPI1, a repressor of erythroid differentiation (Hoppe *et al.*, 2016), as the most significant TF. Interestingly, except for BCL11A and PIK3CG, most of the targets of SPI1 in the gene set, including BPI, CD44, LCP1 and PTPRC, were downregulated (see Supplementary Fig. S5). Therefore, excluding the targets of SPI1 and considering only TFs, we arrived with BATF as the most interesting candidate gene regulating T2EC differentiation. BATF is a member of the family of basic leucine zipper transcription factors, and has known function in the development of numerous cell types involved in the immune response (Murphy *et al.*, 2013). But, the possible role of BATF in regulating erythroid differentiation has not been previously reported. An experimental confirmation of this finding is currently underway.

### 3.4 Computational runtimes

To assess the computational complexity of our approach, we measured the runtimes of SINCERITIES for 10- and 20-gene *in silico* datasets, and compared these runtimes to those of TSNI, GENIE3 and JUMP3. Table 4 gives the average runtimes (in seconds) for these methods for the main *in silico* dataset and for THP-1 differentiation data. Tree-based inference methods (GENIE3 and JUMP3) were significantly slower than SINCERITIES and TSNI. In particular, doubling the network size, the runtimes of GENIE3 and JUMP3 doubled and quadrupled, respectively. Meanwhile, the runtimes of SINCERITIES and TSNI finished almost instantaneously (<1 s) for these datasets, since these algorithms involved solving linear regressions. Finally, we noted that the regularized linear regressions in SINCERITIES are independent of each other and are therefore amenable for parallel computation.

**Table 4.** Computational times comparison among TSNI, GENIE3, JUMP3 and SINCERITIES

Average runtime (*)	TSNI	GENIE3	JUMP3	SINCERITIES
10-gene networks	0.04 s	16 s	6 s	0.32 s
20-gene networks	0.06 s	40 s	24 s	0.74 s
THP-1 differentiation data	0.33 s	41 s	43 s	0.83 s

(*) All timings were measured on an 8-GB RAM, 1.6 GHz dual-core Intel core i5 computer.

## 4 Discussion

Advances in single cell transcriptional profiling offer much promise in elucidating the functional role of cell-to-cell variability across different key physiological processes, such as stem cell differentiation. In particular, single cell expression data carry crucial information on the gene regulatory network that governs cellular heterogeneity and cell decision-making. The challenges of analyzing single cell transcriptional data have led to the creation of novel bioinformatics algorithms, including algorithms for GRN inference using single cell transcriptional profiles (Chen *et al.*, 2015; Matsumoto *et al.*, 2017; Moignard *et al.*, 2015; Ocone *et al.*, 2015; Pina *et al.*, 2015). However, the prediction of gene-gene interactions from single cell transcriptional profiles is complicated by the intrinsic stochasticity and bursty dynamics of the gene expression process and the loss of cell identity during high-throughput transcriptional profiling.

A number of algorithms have been developed based on viewing the single cell gene expressions as binary state vectors, whose state transition trajectories are governed by a gene regulatory network with Boolean logic functions. Examples of such algorithms include SCNS (Moignard *et al.*, 2015), SingleCellNet (Chen *et al.*, 2015) and BTR (Lim *et al.*, 2016). A general drawback of these algorithms is that the dimension of the state space of a Boolean network increases exponentially with respect to the number of genes (2^*m*^ where *m* is the number of genes). Consequently, even for a moderately sized GRN (∼50 genes), providing a reasonable coverage of the state space would require a tremendous number of single cell profiles. The extremely large state space will also make the inference problem computationally challenging.

Recently, Ocone et al. used a combination of a machine-learning algorithm GENIE3 and ODE modelling for GRN inference using single cell transcriptional data (Ocone *et al.*, 2015). Here, GENIE3 was first applied to produce a skeleton of the GRN. This skeleton was then refined by fitting an ODE model to pseudo-time trajectories of the gene expression, produced by applying Wanderlust algorithm to single cell expression data in low-dimensional diffusion map projection (Coifman and Lafon, 2006). However, there are several issues in using pseudo-time trajectories for GRN inference. First, one makes an implicit assumption that the trajectory reflects the gene expression changes resulting from the gene regulatory interactions during the physiological process of interest (e.g. cell differentiation). The pseudo-time approach further assumes that the transition between cell states is deterministic, a hypothesis that is still hotly debated (Moris *et al.*, 2016).

In our experience, the success of cell ordering in reproducing the gene expression trajectory depends sensitively on the cell sampling strategy, that is, being able to sample the right cells at the right time point or stages. For example, the application of Wanderlust to the *in silico* time-stamped single cell dataset from yeast led to cell ordering that was incongruent with the sampling times, especially for latter time points (see Supplementary Figs S1 and S2). Meanwhile, Kouno et al. showed by using multiple dimension scaling that the THP-1 cell differentiation follows a rather irregular temporal dynamics in the low-dimensional (2D) projected space (see also Supplementary Fig. S3 for PCA, t-SNE and diffusion map analysis). As in the case of *in silico* dataset, Wanderlust ordering of THP-1 single cell expression data showed little correlation with the cell time stamps (see Supplementary Fig. S4). A similar situation was also reported for the T2EC dataset, where cell ordering using several pseudo-time algorithms led to incongruous outcomes (Richard *et al.*, 2016). But, if the pseudo-time of the single cells could be generated, SINCERITIES could still be used with the pseudo-time replacing the time stamp. For this purpose, the cells should be first binned according to their pseudo-times. Subsequently, one can apply SINCERITIES using the pseudo-times of bin centers as the time stamps. Such a strategy is particularly appropriate when the cell differentiation progression is asynchronous.

SINCERITIES could overcome the issues of large dataset requirement, high computational complexity, and difficult cell ordering, as the network inference involves numerically efficient regularized linear regression and directly use time-stamped cross-sectional data. SINCERITIES relies on the dynamical changes in the gene expression distributions via DDs to establish a directed GRN graph based on the Granger causality concept. Here, the directed edges imply a predictive causality, where the DDs of the regulators over a given time window have the information for predicting the DDs of the target gene one time window ahead. In comparison to our previous algorithm SNIFS (Sparse Network Inference For Single cell data) that employed Lasso (Papili Gao *et al.*, 2016), SINCERITIES provides predictions for the mode of the gene regulations (i.e. the sign of the edges), and accommodates unevenly spaced time points—a common characteristic of time-stamped single cell datasets. When the time intervals are short, SINCERITIES formulation may miss gene regulations due to delayed gene responses. However, as the time windows in the single cell analysis typically differ by hours, such an issue may not be prominent. In addition, given enough time points, one could modify the GRN inference to include additional DDs beyond one time window lag.

SINCERITIES produces a ranked list of edges based on the values of the coefficients *α.* Many GRN inference algorithms generate similar outputs, including TSNI, GENIE3 and JUMP3. As mentioned earlier, the magnitude of *α* coefficients indicates the confidence that a regulatory interaction exists. We allow the end-users to decide the cut-off value for *α* above which regulatory edges should be included in the GRN. Here, one could adopt a variable selection procedure, where the regulatory edges are added sequentially in decreasing magnitude of *α* coefficients, until a pre-selected criterion is satisfied. Examples of such a criterion include Akaike information criterion, Bayesian information criterion and Mallow’s Cp criterion (Yanagihara and Satoh, 2010).

Finally, SINCERITIES formulation in Eq. (1) does not include any combinatorial regulatory interactions—the regulation of the expression of a gene by two or more regulators together. To account for such combinatorial regulations, one could modify the linear regression problem to include the time-changes in the joint gene expression distribution of multiple regulators among the set of regressors [i.e. in the right hand side of Eq. (1)]. For this purpose, one could use the multi-dimensional extension of KS distance (Fasano and Franceschini, 1987; Justel *et al.*, 1997). The computational cost of performing ridge regression would obviously increase, an issue that could be mitigated using parallel computing (through GLMNET parallel option). However, the calculation of KS distances beyond bivariate distributions (i.e. more than two regulators) poses a considerable algorithmic challenge, for which several numerically efficient approximations have been proposed (Fasano and Franceschini, 1987; Justel *et al.*, 1997; Xiao, 2017).

## 5 Conclusion

In this work, we developed SINCERITIES for GRN inference using time-stamped cross-sectional single cell expression data, a common type of dataset generated by transcriptional profiling of single cells at multiple time points. SINCERITIES is based on the premise that changes in the gene expression distribution of a transcription factor in a given time window would cause a proportional change in the transcriptional expression distributions of the target genes in the next time window. The network inference involves numerically efficient ridge regression problem. In comparison to network inference algorithms for population average time series data (TSNI and JUMP3) and to a tree-based machine learning algorithm (GENIE3), SINCERITIES could provide significantly more accurate GRNs based on AUROCs and AUPRs.

## Supplementary Material

Supplementary Data
