# Supplementary material for: SINCERITIES: inferring gene regulatory networks from time-stamped single cell transcriptional expression profiles
Source: Bioinformatics. 2017 Sep 14;34(2):258–66. doi: 10.1093/bioinformatics/btx575 (PMC5860204; doi:10.1093/bioinformatics/btx575)

Network E. coli 1	G1	G2	G3	G4	G5	G6	G7	G8	G9	G10
G1 →	0	-1	0	0	0	0	0	0	0	0
G2 →	0	0	0	0	0	0	1	0	0	0
G3 →	-1	0	0	1	-1	0	0	-1	0	-1
G4 →	0	0	0	0	0	0	0	0	0	0
G5 →	0	0	0	0	0	0	-1	0	-1	0
G6 →	0	0	1	0	0	0	0	1	0	1
G7 →	0	0	0	0	0	0	0	0	0	0
G8 →	0	0	0	0	0	0	0	0	0	0
G9 →	0	0	0	0	0	0	0	0	0	0
G10 →	0	0	0	0	0	0	0	0	0	0

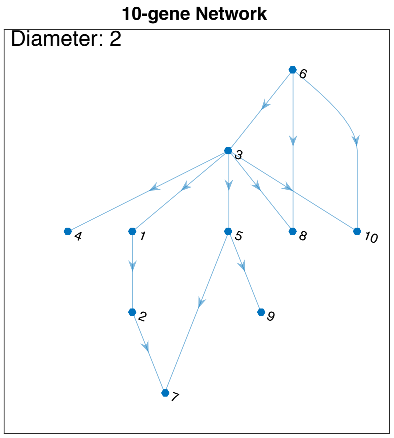

Network E. coli 2	G1	G2	G3	G4	G5	G6	G7	G8	G9	G10
G1 →	0	-1	0	0	0	0	0	0	0	0
G2 →	0	0	0	0	0	0	0	0	0	0
G3 →	0	0	0	1	1	1	1	1	1	-1
G4 →	0	0	0	0	0	0	0	0	0	0
G5 →	0	0	0	0	0	0	0	0	0	0
G6 →	0	0	0	0	0	0	0	-1	0	0
G7 →	0	0	0	0	0	0	0	0	0	0
G8 →	0	0	0	0	0	0	0	0	0	0
G9 →	0	0	0	0	0	0	0	0	0	0
G10 →	0	0	0	0	0	0	0	0	0	0

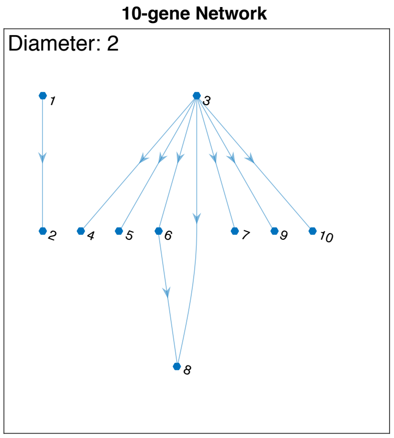

Network E. coli 3	G1	G2	G3	G4	G5	G6	G7	G8	G9	G10
G1 →	0	0	0	0	0	0	1	0	0	0
G2 →	-1	0	0	0	0	0	0	0	0	0
G3 →	-1	0	0	0	0	0	0	0	0	0
G4 →	1	0	0	0	0	0	0	0	0	0
G5 →	-1	0	0	0	0	-1	0	1	1	0
G6 →	0	0	0	0	0	0	0	0	0	0
G7 →	0	0	0	0	0	0	0	0	0	0
G8 →	0	0	0	0	0	0	0	0	0	0
G9 →	0	0	0	0	0	0	0	0	0	0
G10 →	0	0	0	0	0	0	0	0	0	0

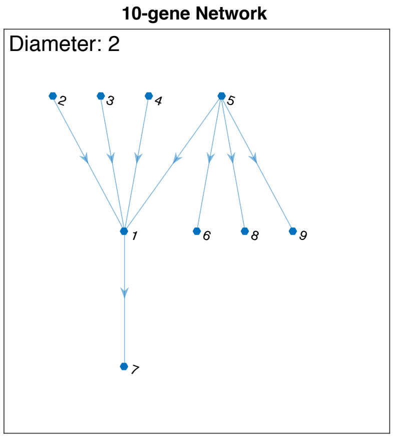

Network E. coli 4	G1	G2	G3	G4	G5	G6	G7	G8	G9	G10
G1 →	0	-1	-1	0	-1	-1	-1	-1	-1	-1
G2 →	0	0	0	0	0	0	0	1	0	0
G3 →	0	1	0	0	0	0	1	1	1	0
G4 →	1	0	0	0	0	0	0	0	0	0
G5 →	0	0	0	0	0	0	0	0	0	0
G6 →	0	0	0	0	0	0	0	0	0	0
G7 →	0	0	0	0	0	0	0	0	0	0
G8 →	0	0	0	0	0	0	0	0	0	0
G9 →	0	0	0	0	0	0	0	0	0	0
G10 →	0	0	0	0	0	0	0	0	0	0

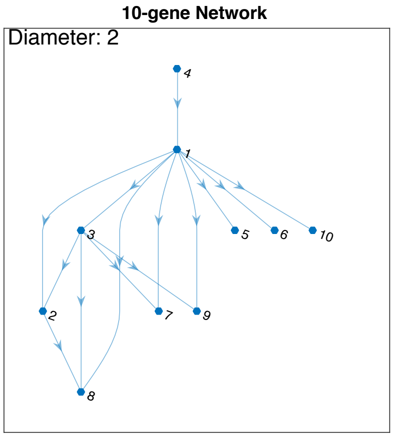

Network E. coli 5	G1	G2	G3	G4	G5	G6	G7	G8	G9	G10
G1 →	0	0	0	0	0	0	1	0	0	0
G2 →	1	0	0	0	0	0	-1	0	0	0
G3 →	1	0	0	1	0	0	0	0	0	0
G4 →	0	0	0	0	-1	0	0	0	0	0
G5 →	0	0	0	0	0	0	0	0	0	0
G6 →	0	0	1	0	0	0	0	1	1	1
G7 →	0	0	0	0	0	0	0	0	0	0
G8 →	0	0	0	0	0	0	0	0	0	0
G9 →	0	0	0	0	0	0	0	0	0	0
G10 →	0	0	0	0	0	0	0	0	0	0

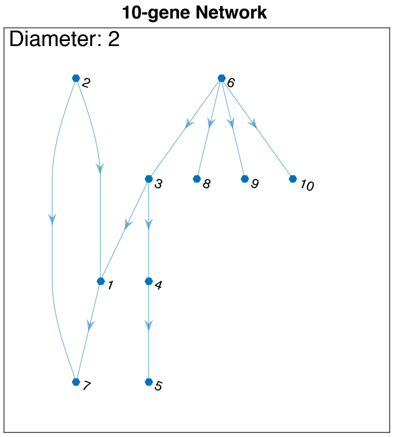

Network E. coli 6	G1	G2	G3	G4	G5	G6	G7	G8	G9	G10
G1 →	0	1	0	0	0	0	0	0	0	0
G2 →	0	0	-1	-1	0	1	-1	-1	0	-1
G3 →	0	0	0	0	0	0	0	0	0	0
G4 →	0	0	1	0	-1	0	1	0	1	0
G5 →	0	0	0	0	0	0	0	0	0	0
G6 →	0	0	0	0	0	0	0	0	0	0
G7 →	0	0	0	0	0	0	0	0	0	0
G8 →	0	0	0	0	0	0	0	0	0	0
G9 →	0	0	0	0	0	0	0	0	0	0
G10 →	0	0	0	0	0	0	0	0	0	0

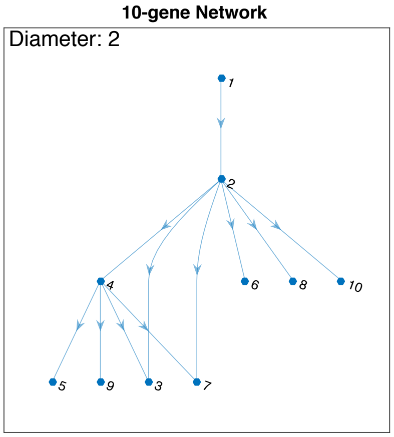

Network E. coli 7	G1	G2	G3	G4	G5	G6	G7	G8	G9	G10
G1 →	0	1	1	0	0	0	1	1	1	1
G2 →	0	0	-1	0	0	0	-1	0	0	0
G3 →	-1	1	0	0	1	0	1	1	0	1
G4 →	0	1	1	0	0	0	1	1	1	1
G5 →	0	0	0	0	0	0	0	0	0	0
G6 →	0	0	0	1	0	0	0	1	0	0
G7 →	0	0	0	0	0	0	0	0	0	0
G8 →	0	0	0	0	0	0	0	0	0	0
G9 →	0	0	0	0	0	0	0	0	0	0
G10 →	0	0	0	0	0	0	0	0	0	0

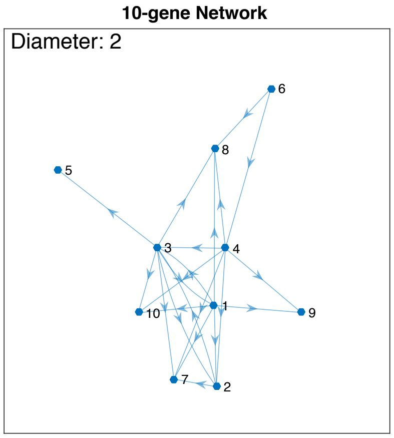

Network E. coli 8	G1	G2	G3	G4	G5	G6	G7	G8	G9	G10
G1 →	0	0	0	0	0	0	0	0	0	0
G2 →	-1	0	-1	-1	0	0	0	0	-1	-1
G3 →	0	0	0	0	0	0	0	0	0	0
G4 →	0	0	0	0	0	0	0	0	0	0
G5 →	0	1	0	0	0	0	0	0	-1	1
G6 →	-1	0	0	0	0	0	0	1	0	0
G7 →	0	0	0	0	0	0	0	0	0	0
G8 →	0	0	0	0	0	0	0	0	0	0
G9 →	0	0	0	0	0	0	0	0	0	0
G10 →	0	0	0	0	0	0	0	0	0	0

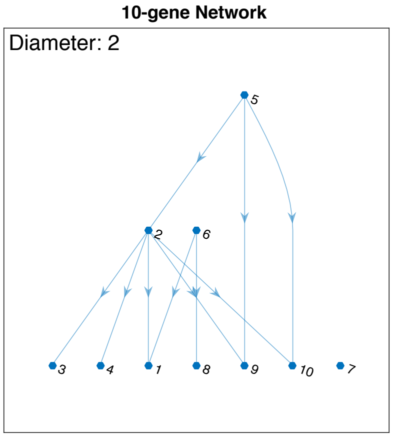

Network E. coli 9	G1	G2	G3	G4	G5	G6	G7	G8	G9	G10
G1 →	0	0	0	0	0	0	1	0	0	0
G2 →	1	0	-1	0	0	0	1	1	-1	1
G3 →	0	0	0	0	0	0	0	0	1	0
G4 →	0	0	-1	0	0	0	0	0	0	0
G5 →	0	0	1	0	0	0	0	0	0	0
G6 →	0	0	-1	0	0	0	0	0	0	0
G7 →	0	0	0	0	0	0	0	0	0	0
G8 →	0	0	0	0	0	0	0	0	0	0
G9 →	0	0	0	0	0	0	0	0	0	0
G10 →	0	0	0	0	0	0	0	0	0	0

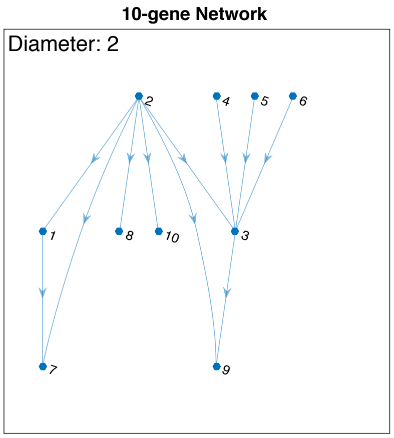

Network E. coli 10	G1	G2	G3	G4	G5	G6	G7	G8	G9	G10
G1 →	0	-1	-1	-1	0	0	-1	0	0	-1
G2 →	0	0	0	0	0	0	0	0	0	0
G3 →	0	0	0	0	0	0	0	0	0	0
G4 →	0	0	0	0	0	0	0	0	0	0
G5 →	1	0	0	0	0	0	1	0	0	1
G6 →	0	0	0	-1	0	0	0	-1	-1	0
G7 →	0	0	0	0	0	0	0	0	0	0
G8 →	0	0	0	0	0	0	0	0	0	0
G9 →	0	0	0	0	0	0	0	0	0	0
G10 →	0	0	0	0	0	0	0	0	0	0

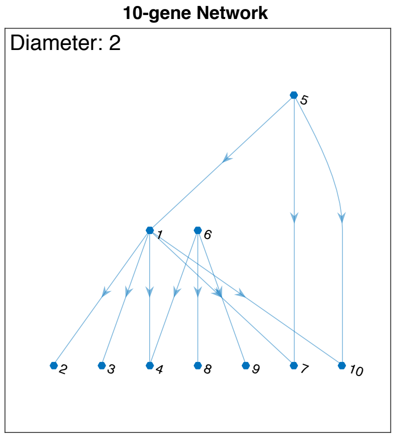

Network Yeast 1	G1	G2	G3	G4	G5	G6	G7	G8	G9	G10
G1 →	0	0	1	0	0	0	1	1	1	1
G2 →	1	0	0	0	0	0	0	0	0	0
G3 →	0	0	0	0	0	0	0	0	0	0
G4 →	0	0	1	0	1	0	1	0	0	0
G5 →	0	0	0	0	0	1	0	0	0	0
G6 →	0	0	0	0	0	0	0	0	0	1
G7 →	0	0	0	0	0	0	0	0	0	0
G8 →	0	0	0	0	0	0	0	0	0	0
G9 →	0	0	0	0	0	0	0	0	0	0
G10 →	0	0	0	0	0	0	0	0	0	0

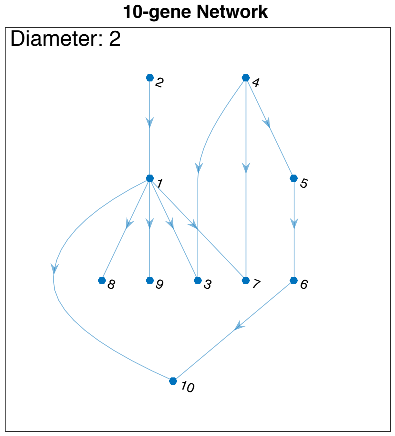

Network Yeast 2	G1	G2	G3	G4	G5	G6	G7	G8	G9	G10
G1 →	0	1	1	0	0	0	1	1	1	1
G2 →	0	0	0	0	0	0	0	0	0	0
G3 →	0	0	0	0	0	0	0	0	0	0
G4 →	0	0	0	0	0	0	0	0	0	0
G5 →	0	0	0	1	0	0	0	0	0	0
G6 →	0	0	0	1	0	0	0	0	0	0
G7 →	0	0	0	0	0	0	0	0	0	0
G8 →	0	0	0	0	0	0	0	0	0	0
G9 →	0	0	0	0	0	0	0	0	0	0
G10 →	0	0	0	0	0	0	0	0	0	0

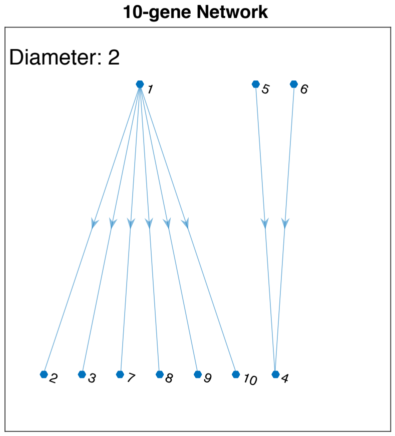

Network Yeast 3	G1	G2	G3	G4	G5	G6	G7	G8	G9	G10
G1 →	0	1	0	0	0	0	0	0	1	0
G2 →	0	0	0	0	0	0	0	1	0	1
G3 →	0	1	0	0	0	0	0	0	0	0
G4 →	0	1	0	0	0	0	1	0	1	0
G5 →	0	1	0	0	0	1	1	1	0	0
G6 →	0	0	0	0	0	0	0	0	0	0
G7 →	0	0	0	0	0	0	0	0	0	0
G8 →	0	0	0	0	0	0	0	0	0	0
G9 →	0	0	0	0	0	0	0	0	0	0
G10 →	0	0	0	0	0	0	0	0	0	0

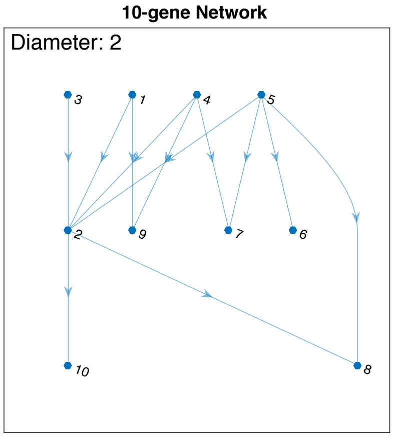

Network Yeast 4	G1	G2	G3	G4	G5	G6	G7	G8	G9	G10
G1 →	0	1	1	0	0	0	1	1	1	0
G2 →	0	0	0	0	0	0	0	0	0	1
G3 →	0	0	0	0	0	1	0	0	0	0
G4 →	0	1	1	0	0	0	1	1	1	0
G5 →	0	1	0	0	0	0	1	1	0	0
G6 →	0	0	0	0	0	0	0	0	0	0
G7 →	0	0	0	0	0	0	0	0	0	0
G8 →	0	0	0	0	0	0	0	0	0	0
G9 →	0	0	0	0	0	0	0	0	0	0
G10 →	0	0	0	0	0	0	0	0	0	0

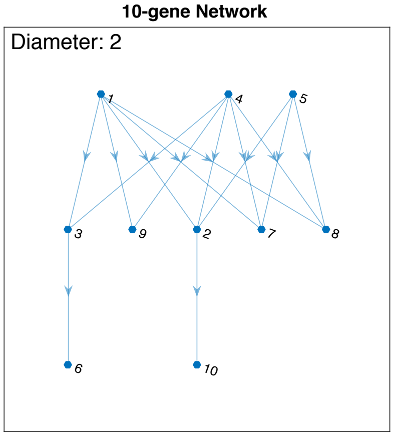

Network Yeast 5	G1	G2	G3	G4	G5	G6	G7	G8	G9	G10
G1 →	0	1	0	0	0	0	1	0	1	1
G2 →	0	0	1	1	0	0	0	0	0	0
G3 →	0	0	0	0	0	0	0	0	0	0
G4 →	0	0	0	0	0	0	0	1	0	0
G5 →	0	0	0	1	0	0	0	0	0	0
G6 →	0	0	0	1	1	0	1	0	0	1
G7 →	0	0	0	0	0	0	0	0	0	0
G8 →	0	0	0	0	0	0	0	0	0	0
G9 →	0	0	0	0	0	0	0	0	0	0
G10 →	0	0	0	0	0	0	0	0	0	0

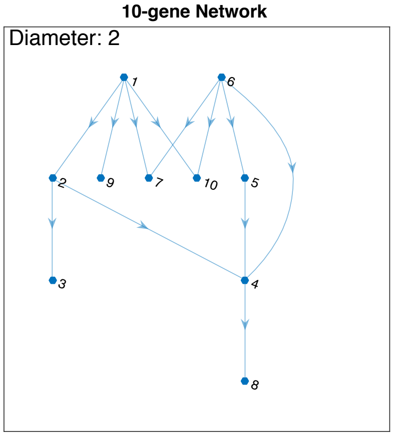

Network Yeast 6	G1	G2	G3	G4	G5	G6	G7	G8	G9	G10
G1 →	0	1	0	0	0	0	0	0	0	0
G2 →	0	0	0	0	0	0	0	0	0	0
G3 →	0	1	0	1	0	0	0	0	0	0
G4 →	0	0	0	0	0	0	0	0	0	0
G5 →	0	1	0	0	0	0	0	1	0	1
G6 →	0	0	0	1	0	0	1	1	1	1
G7 →	0	0	0	0	0	0	0	0	0	0
G8 →	0	0	0	0	0	0	0	0	0	0
G9 →	0	0	0	0	0	0	0	0	0	0
G10 →	0	0	0	0	0	0	0	0	0	0

10-gene Network

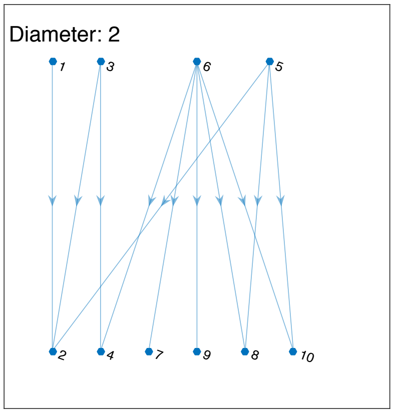

Network Yeast 7	G1	G2	G3	G4	G5	G6	G7	G8	G9	G10
G1 →	0	1	0	0	0	0	1	1	1	1
G2 →	0	0	0	0	0	0	0	0	0	0
G3 →	0	1	0	1	0	0	0	0	0	0
G4 →	0	0	0	0	0	0	0	0	0	0
G5 →	0	0	0	0	0	0	0	0	0	0
G6 →	0	1	0	0	1	0	0	0	0	1
G7 →	0	0	0	0	0	0	0	0	0	0
G8 →	0	0	0	0	0	0	0	0	0	0
G9 →	0	0	0	0	0	0	0	0	0	0
G10 →	0	0	0	0	0	0	0	0	0	0

10-gene Network

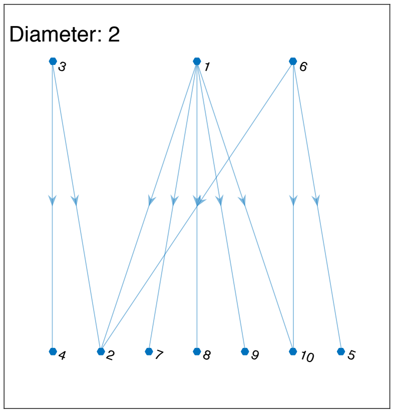

Network Yeast 8	G1	G2	G3	G4	G5	G6	G7	G8	G9	G10
G1 →	0	1	1	0	0	0	1	1	0	1
G2 →	0	0	0	0	0	0	0	0	0	0
G3 →	0	0	0	1	0	0	0	0	0	0
G4 →	0	0	0	0	0	0	0	0	0	0
G5 →	0	0	1	1	0	0	0	0	1	0
G6 →	0	0	1	1	0	0	1	1	1	1
G7 →	0	0	0	0	0	0	0	0	0	0
G8 →	0	0	0	0	0	0	0	0	0	0
G9 →	0	0	0	0	0	0	0	0	0	0
G10 →	0	0	0	0	0	0	0	0	0	0

10-gene Network

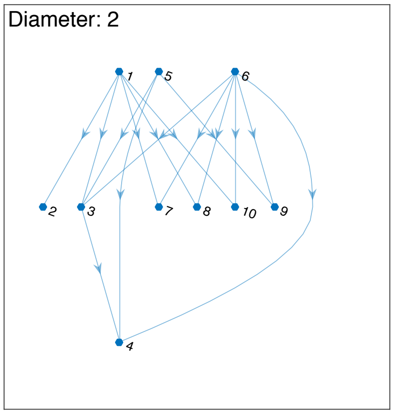

Network Yeast 9	G1	G2	G3	G4	G5	G6	G7	G8	G9	G10
G1 →	0	1	0	0	1	0	1	0	0	1
G2 →	0	0	0	0	0	1	0	1	0	0
G3 →	0	1	0	0	0	0	1	0	1	1
G4 →	0	1	0	0	0	0	0	0	1	0
G5 →	0	0	0	0	0	0	0	0	0	0
G6 →	0	0	0	0	0	0	0	0	0	0
G7 →	0	0	0	0	0	0	0	0	0	0
G8 →	0	0	0	0	0	0	0	0	0	0
G9 →	0	0	0	0	0	0	0	0	0	0
G10 →	0	0	0	0	0	0	0	0	0	0

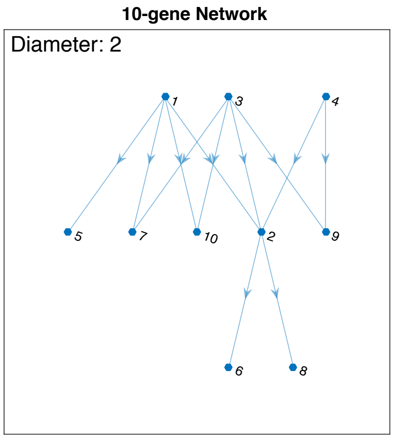

Network Yeast 10	G1	G2	G3	G4	G5	G6	G7	G8	G9	G10
G1 →	0	0	0	0	0	0	0	0	0	0
G2 →	1	0	1	0	0	0	1	0	0	1
G3 →	0	0	0	0	1	0	0	0	0	0
G4 →	1	0	1	0	0	0	0	1	1	1
G5 →	0	0	0	0	0	0	0	0	0	0
G6 →	1	0	0	0	0	0	1	1	1	1
G7 →	0	0	0	0	0	0	0	0	0	0
G8 →	0	0	0	0	0	0	0	0	0	0
G9 →	0	0	0	0	0	0	0	0	0	0
G10 →	0	0	0	0	0	0	0	0	0	0

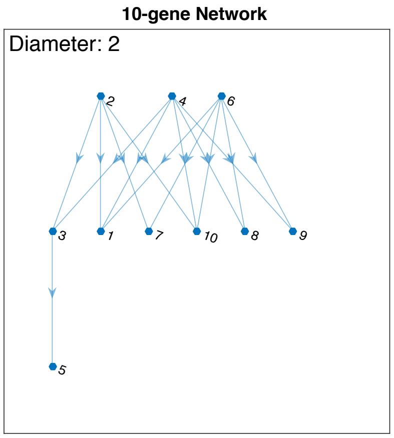

Network E. coli 1	G1	G2	G3	G4	G5	G6	G7	G8	G9	G10	G11	G12	G13	G14	G15	G16	G17	G18	G19	G20
G1 →	0	-1	-1	1	0	0	0	0	0	0	0	0	0	0	-1	0	0	-1	0	-1
G2 →	0	0	0	0	0	0	0	0	0	0	0	0	0	0	0	0	0	0	0	0
G3 →	0	0	0	0	0	0	0	0	0	0	0	0	0	0	0	0	0	0	0	-1
G4 →	0	0	0	0	0	0	0	0	0	0	0	0	0	0	0	0	0	0	0	0
G5 →	0	0	0	0	0	0	0	-1	0	1	0	0	1	1	1	1	1	1	1	0
G6 →	0	0	0	0	-1	0	0	0	0	0	0	0	1	0	0	0	0	0	0	0
G7 →	0	0	0	0	-1	0	0	-1	-1	0	0	0	0	0	0	0	0	0	0	0
G8 →	0	0	0	0	-1	0	0	0	0	1	0	0	1	1	0	0	1	0	-1	0
G9 →	0	0	0	0	0	0	0	0	0	0	0	0	0	0	0	0	0	0	0	0
G10 →	0	0	0	0	1	0	0	1	0	0	0	0	1	0	1	1	1	1	1	0
G11 →	0	0	0	0	0	0	0	0	0	1	0	1	0	0	0	0	1	0	0	0
G12 →	0	0	0	0	0	0	0	0	0	1	0	0	0	0	0	0	1	0	0	0
G13 →	0	0	0	0	0	0	0	0	0	0	0	0	0	0	0	0	0	0	0	0
G14 →	0	0	0	0	0	0	0	0	0	0	0	0	0	0	0	0	0	0	0	0
G15 →	0	0	0	0	0	0	0	0	0	0	0	0	0	0	0	0	0	0	0	0
G16 →	0	0	0	0	0	0	0	0	0	0	0	0	0	0	0	0	0	0	0	0
G17 →	0	0	0	0	0	0	0	0	0	0	0	0	0	0	0	0	0	0	0	0
G18 →	0	0	0	0	0	0	0	0	0	0	0	0	0	0	0	0	0	0	0	0
G19 →	0	0	0	0	0	0	0	0	0	0	0	0	0	0	0	0	0	0	0	0
G20 →	0	0	0	0	0	0	0	0	0	0	0	0	0	0	0	0	0	0	0	0

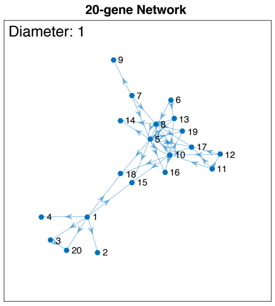

Network E. coli 2	G1	G2	G3	G4	G5	G6	G7	G8	G9	G10	G11	G12	G13	G14	G15	G16	G17	G18	G19	G20
G1 →	0	0	0	0	0	0	0	0	0	0	0	0	0	0	0	0	0	0	0	0
G2 →	0	0	0	0	0	0	0	0	0	0	0	0	0	0	0	0	0	0	0	0
G3 →	0	0	0	-1	-1	1	1	0	-1	1	-1	0	0	0	0	0	0	0	1	0
G4 →	0	0	0	0	0	0	0	0	0	0	0	0	0	0	0	0	0	0	0	0
G5 →	0	0	0	0	0	0	0	0	0	0	0	0	0	0	0	0	0	0	0	0
G6 →	0	0	0	0	0	0	-1	0	0	0	0	0	0	0	0	0	0	0	0	0
G7 →	0	0	0	0	0	1	0	1	0	0	0	0	0	0	1	1	1	1	0	1
G8 →	0	0	0	0	0	0	0	0	0	0	0	0	0	0	0	0	0	0	0	0
G9 →	0	0	0	0	0	0	0	0	0	0	0	0	0	0	0	0	0	0	0	0
G10 →	0	0	0	0	0	0	0	0	0	0	0	0	-1	-1	0	0	0	0	0	0
G11 →	0	0	0	0	0	0	0	0	0	0	0	0	-1	-1	0	0	0	0	0	0
G12 →	0	0	0	0	0	1	1	0	0	0	0	0	0	0	1	1	1	1	0	1
G13 →	0	0	0	0	0	0	0	0	0	0	0	0	0	0	0	0	0	0	0	0
G14 →	0	0	0	0	0	0	0	0	0	0	0	0	0	0	0	0	0	0	0	0
G15 →	0	0	0	0	0	0	0	0	0	0	0	0	0	0	0	0	0	0	0	0
G16 →	0	0	0	0	0	0	0	0	0	0	0	0	0	0	0	0	0	0	0	0
G17 →	0	0	0	0	0	0	0	0	0	0	0	0	0	0	0	0	0	0	0	0
G18 →	0	0	0	0	0	0	0	0	0	0	0	0	0	0	0	0	0	0	0	0
G19 →	0	0	0	0	0	0	0	0	0	0	0	0	0	0	0	0	0	0	0	0
G20 →	0	0	0	0	0	0	0	0	0	0	0	0	0	0	0	0	0	0	0	0

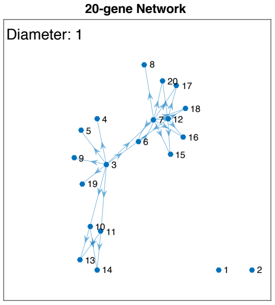

Network E. coli 3	G1	G2	G3	G4	G5	G6	G7	G8	G9	G10	G11	G12	G13	G14	G15	G16	G17	G18	G19	G20
G1 →	0	1	0	0	0	0	0	0	0	0	0	0	0	0	0	0	1	1	0	0
G2 →	-1	0	0	0	0	0	0	0	0	0	0	0	0	0	0	0	-1	-1	0	0
G3 →	1	1	0	0	-1	-1	-1	-1	-1	-1	0	-1	-1	0	-1	-1	1	1	-1	-1
G4 →	0	0	1	0	0	0	0	0	0	0	0	0	0	0	0	0	0	0	0	0
G5 →	0	0	0	0	0	0	0	0	0	0	0	0	0	0	0	0	0	0	0	0
G6 →	0	0	0	0	0	0	0	0	0	0	0	0	0	1	0	0	0	0	1	0
G7 →	0	0	0	0	0	0	0	0	0	0	0	0	0	0	0	0	0	0	0	0
G8 →	0	0	0	0	0	0	1	0	0	0	-1	0	0	0	0	0	0	0	0	0
G9 →	0	0	0	0	0	0	0	0	0	0	0	0	0	0	0	0	0	0	0	0
G10 →	0	0	0	0	0	0	0	0	0	0	0	0	0	0	0	0	0	0	0	0
G11 →	0	0	0	0	0	0	0	0	0	0	0	0	0	0	0	1	0	0	0	0
G12 →	0	0	0	0	0	0	0	0	0	0	0	0	0	0	0	0	0	0	0	0
G13 →	0	0	0	0	0	0	0	0	0	0	0	0	0	0	0	0	0	0	0	0
G14 →	0	0	0	0	0	0	0	0	0	0	0	0	0	0	0	0	0	0	0	0
G15 →	0	0	0	0	0	0	0	0	0	0	0	0	0	0	0	0	0	0	0	0
G16 →	0	0	0	0	0	0	0	0	0	0	0	0	0	0	0	0	0	0	0	0
G17 →	0	0	0	0	0	0	0	0	0	0	0	0	0	0	0	0	0	0	0	0
G18 →	0	0	0	0	0	0	0	0	0	0	0	0	0	0	0	0	0	0	0	0
G19 →	0	0	0	0	0	0	0	0	0	0	0	0	0	0	0	0	0	0	0	0
G20 →	0	0	0	0	0	0	0	0	0	0	0	0	0	0	0	0	0	0	0	0

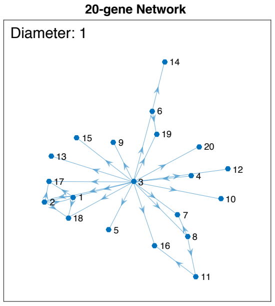

Network E. coli 4	G1	G2	G3	G4	G5	G6	G7	G8	G9	G10	G11	G12	G13	G14	G15	G16	G17	G18	G19	G20
G1 →	0	0	0	0	0	0	0	0	0	0	0	0	0	0	0	0	0	0	0	0
G2 →	1	0	-1	0	0	0	0	0	0	0	0	0	0	1	1	0	0	1	1	0
G3 →	0	0	0	0	0	0	0	0	0	0	0	0	0	0	0	0	0	0	0	0
G4 →	-1	-1	0	0	-1	-1	-1	1	0	-1	-1	1	1	0	0	0	0	-1	0	-1
G5 →	0	0	0	0	0	0	0	0	0	0	0	0	0	0	0	0	1	0	0	0
G6 →	0	0	0	0	0	0	0	0	0	0	0	0	0	0	0	0	0	-1	0	0
G7 →	0	0	0	0	0	0	0	0	0	0	0	0	0	0	0	1	0	0	0	0
G8 →	0	0	0	0	0	0	0	0	0	0	0	-1	-1	0	0	0	0	0	0	0
G9 →	0	0	0	1	0	0	0	0	0	0	0	0	0	0	0	0	0	0	0	0
G10 →	0	0	0	0	0	0	0	0	0	0	0	0	0	0	0	0	0	0	0	0
G11 →	0	0	0	0	0	0	0	0	0	0	0	0	0	0	0	0	0	0	0	0
G12 →	0	0	0	0	0	0	0	1	0	0	0	0	1	0	0	0	0	0	0	0
G13 →	0	0	0	0	0	0	0	0	0	0	0	0	0	0	0	0	0	0	0	0
G14 →	0	0	0	0	0	0	0	0	0	0	0	0	0	0	0	0	0	0	0	0
G15 →	0	0	0	0	0	0	0	0	0	0	0	0	0	0	0	0	0	0	0	0
G16 →	0	0	0	0	0	0	0	0	0	0	0	0	0	0	0	0	0	0	0	0
G17 →	0	0	0	0	0	0	0	0	0	0	0	0	0	0	0	0	0	0	0	0
G18 →	0	0	0	0	0	0	0	0	0	0	0	0	0	0	0	0	0	0	0	0
G19 →	0	0	0	0	0	0	0	0	0	0	0	0	0	0	0	0	0	0	0	0
G20 →	0	0	0	0	0	0	0	0	0	0	0	0	0	0	0	0	0	0	0	0

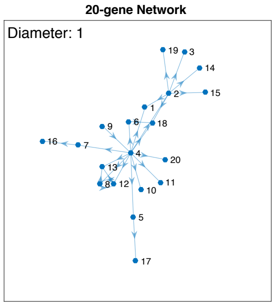

Network E. coli 5	G1	G2	G3	G4	G5	G6	G7	G8	G9	G10	G11	G12	G13	G14	G15	G16	G17	G18	G19	G20
G1 →	0	0	0	0	0	0	0	0	0	0	0	0	0	0	0	0	0	0	0	0
G2 →	-1	0	1	0	0	0	0	0	0	0	0	0	0	0	0	0	0	1	1	0
G3 →	0	0	0	-1	0	0	0	0	0	0	0	0	-1	0	0	0	0	0	0	0
G4 →	0	0	0	0	0	0	0	0	0	0	0	0	1	1	0	0	0	0	0	0
G5 →	0	0	0	1	0	-1	0	-1	0	0	0	0	1	0	1	0	0	0	0	0
G6 →	0	0	0	0	0	0	0	0	0	0	0	0	0	0	0	0	0	0	0	0
G7 →	0	0	0	-1	0	0	0	-1	0	0	1	0	-1	0	0	1	1	0	0	1
G8 →	0	0	0	0	0	0	0	0	0	0	0	0	0	0	0	0	0	0	0	0
G9 →	0	0	0	0	0	0	0	-1	0	0	0	0	0	0	0	0	0	0	0	0
G10 →	0	0	0	0	0	0	0	1	0	0	0	0	0	0	0	0	0	0	0	0
G11 →	0	0	0	-1	0	0	0	-1	0	0	0	0	-1	0	0	1	1	0	0	1
G12 →	0	0	0	0	0	0	0	-1	0	0	0	0	0	0	0	0	0	0	0	0
G13 →	0	0	0	0	0	0	0	0	0	0	0	0	0	0	0	0	0	0	0	0
G14 →	0	0	0	0	0	0	0	0	0	0	0	0	0	0	0	0	0	0	0	0
G15 →	0	0	0	0	0	0	0	0	0	0	0	0	0	0	0	0	0	0	0	0
G16 →	0	0	0	0	0	0	0	0	0	0	0	0	0	0	0	0	0	0	0	0
G17 →	0	0	0	0	0	0	0	0	0	0	0	0	0	0	0	0	0	0	0	0
G18 →	0	0	0	0	0	0	0	0	0	0	0	0	0	0	0	0	0	0	0	0
G19 →	0	0	0	0	0	0	0	0	0	0	0	0	0	0	0	0	0	0	0	0
G20 →	0	0	0	0	0	0	0	0	0	0	0	0	0	0	0	0	0	0	0	0

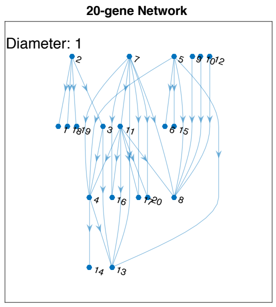

Network E. coli 6	G1	G2	G3	G4	G5	G6	G7	G8	G9	G10	G11	G12	G13	G14	G15	G16	G17	G18	G19	G20
G1 →	0	0	0	0	0	0	0	0	0	0	0	0	0	0	0	1	0	0	0	0
G2 →	1	0	1	1	1	1	1	1	1	1	1	1	-1	1	1	1	1	1	1	1
G3 →	0	0	0	0	0	0	0	0	0	0	0	0	0	0	0	0	0	0	0	0
G4 →	0	0	0	0	0	0	0	0	0	0	0	0	0	0	0	0	0	0	0	0
G5 →	0	0	0	0	0	0	0	0	0	0	0	0	0	0	0	0	0	0	0	0
G6 →	0	0	0	0	0	0	0	1	0	0	0	0	0	0	0	0	0	0	0	0
G7 →	0	0	0	0	0	0	0	0	0	0	0	0	0	0	0	0	0	0	0	0
G8 →	0	0	0	0	0	1	0	0	0	0	0	0	0	0	0	0	0	0	0	0
G9 →	0	0	0	0	0	0	-1	0	0	0	0	0	0	0	0	0	0	0	-1	0
G10 →	0	0	0	0	0	0	0	0	0	0	0	0	0	0	0	0	0	0	0	0
G11 →	0	0	0	0	0	0	0	0	0	0	0	0	0	0	0	0	0	0	0	0
G12 →	0	0	0	0	0	0	0	0	0	0	0	0	0	-1	0	0	0	0	0	0
G13 →	0	0	0	0	0	0	0	0	0	0	0	0	0	0	0	0	0	0	0	0
G14 →	0	0	0	0	0	0	0	0	0	0	0	0	0	0	0	0	0	0	0	0
G15 →	0	0	0	0	0	0	0	0	0	0	0	0	0	0	0	0	0	0	0	0
G16 →	0	0	0	0	0	0	0	0	0	0	0	0	0	0	0	0	0	0	0	0
G17 →	0	0	0	0	0	0	0	0	0	0	0	0	0	0	0	0	0	0	0	0
G18 →	0	0	0	0	0	0	0	0	0	0	0	0	0	0	0	0	0	0	0	0
G19 →	0	0	0	0	0	0	0	0	0	0	0	0	0	0	0	0	0	0	0	0
G20 →	0	0	0	0	0	0	0	0	0	0	0	0	0	0	0	0	0	0	0	0

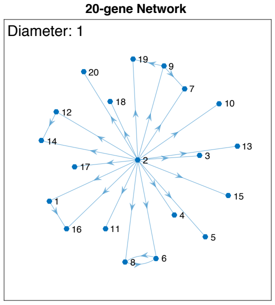

Network E. coli 7	G1	G2	G3	G4	G5	G6	G7	G8	G9	G10	G11	G12	G13	G14	G15	G16	G17	G18	G19	G20
G1 →	0	0	0	0	0	0	0	0	0	0	0	0	0	0	0	0	0	0	0	0
G2 →	-1	0	-1	-1	0	0	0	0	0	0	0	0	0	1	-1	0	-1	-1	-1	-1
G3 →	0	0	0	0	0	0	0	0	0	0	0	0	0	0	-1	0	0	0	0	0
G4 →	0	0	0	0	0	0	0	0	0	0	0	0	0	0	0	0	1	0	0	1
G5 →	0	1	0	0	0	0	0	0	0	0	0	0	0	0	0	0	0	1	-1	0
G6 →	-1	0	0	0	0	0	0	-1	-1	-1	-1	1	1	-1	0	-1	0	0	0	0
G7 →	0	0	0	0	0	1	0	0	0	0	0	0	0	0	0	0	0	0	0	0
G8 →	0	0	0	0	0	0	0	0	0	0	0	0	0	0	0	-1	0	0	0	0
G9 →	0	0	0	0	0	0	0	0	0	0	0	0	0	0	0	0	0	0	0	0
G10 →	0	0	0	0	0	0	0	0	0	0	0	0	0	0	0	0	0	0	0	0
G11 →	0	0	0	0	0	0	0	0	0	0	0	0	0	0	0	0	0	0	0	0
G12 →	0	0	0	0	0	0	0	0	0	0	0	0	0	0	0	0	0	0	0	0
G13 →	0	0	0	0	0	0	0	0	0	0	0	0	0	0	0	0	0	0	0	0
G14 →	0	0	0	0	0	0	0	0	0	0	0	0	0	0	0	0	0	0	0	0
G15 →	0	0	0	0	0	0	0	0	0	0	0	0	0	0	0	0	0	0	0	0
G16 →	0	0	0	0	0	0	0	0	0	0	0	0	0	0	0	0	0	0	0	0
G17 →	0	0	0	0	0	0	0	0	0	0	0	0	0	0	0	0	0	0	0	0
G18 →	0	0	0	0	0	0	0	0	0	0	0	0	0	0	0	0	0	0	0	0
G19 →	0	0	0	0	0	0	0	0	0	0	0	0	0	0	0	0	0	0	0	0
G20 →	0	0	0	0	0	0	0	0	0	0	0	0	0	0	0	0	0	0	0	0

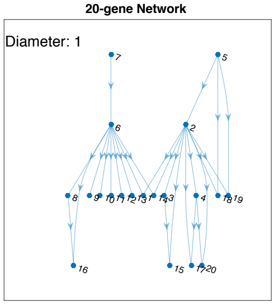

Network E. coli 8	G1	G2	G3	G4	G5	G6	G7	G8	G9	G10	G11	G12	G13	G14	G15	G16	G17	G18	G19	G20
G1 →	0	1	1	1	1	1	1	1	1	1	1	1	1	1	1	1	1	1	1	-1
G2 →	0	0	0	0	0	0	0	0	0	0	0	0	0	0	-1	0	0	0	0	0
G3 →	0	0	0	0	1	0	0	0	0	0	0	0	0	0	0	0	0	0	0	0
G4 →	0	0	0	0	0	0	0	0	0	0	0	0	0	0	0	0	0	0	0	0
G5 →	0	0	1	0	0	0	0	0	0	0	0	0	0	0	0	0	0	0	0	0
G6 →	0	0	0	0	0	0	0	0	0	0	0	0	0	0	0	0	0	0	0	0
G7 →	0	0	0	0	0	0	0	0	0	0	0	0	0	0	0	0	0	0	0	0
G8 →	0	0	0	0	0	0	0	0	0	0	0	0	-1	0	0	0	0	0	0	0
G9 →	0	0	0	0	0	0	0	0	0	0	0	0	0	0	0	0	0	0	0	0
G10 →	0	0	0	0	0	0	0	0	0	0	0	0	0	0	0	0	0	0	0	0
G11 →	0	0	0	0	0	0	0	0	0	0	0	0	0	0	0	0	0	0	0	0
G12 →	0	0	0	0	0	0	0	0	0	0	0	0	0	0	0	0	0	0	0	0
G13 →	0	0	0	0	0	0	0	0	0	0	0	0	0	0	0	0	0	0	0	0
G14 →	0	0	0	0	0	0	0	0	0	0	0	0	0	0	0	0	0	0	0	0
G15 →	0	0	0	0	0	0	0	0	0	0	0	0	0	0	0	0	0	0	0	0
G16 →	0	0	0	0	0	0	0	0	0	0	0	0	0	0	0	0	0	0	0	0
G17 →	0	0	0	0	0	0	0	0	0	0	0	0	0	0	0	0	0	0	0	0
G18 →	0	0	0	0	0	0	0	0	0	0	0	0	0	0	0	0	0	0	0	0
G19 →	0	0	0	0	0	0	0	0	0	0	0	0	0	0	0	0	0	0	0	0
G20 →	0	0	0	0	0	0	0	0	0	0	0	0	0	0	0	0	0	0	0	0

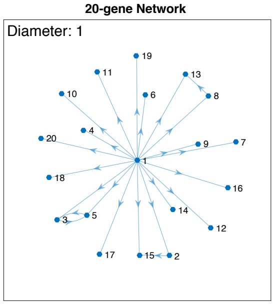

Network E. coli 9	G1	G2	G3	G4	G5	G6	G7	G8	G9	G10	G11	G12	G13	G14	G15	G16	G17	G18	G19	G20
G1 →	0	0	0	0	0	0	0	0	0	0	0	0	0	0	1	0	0	0	0	0
G2 →	1	0	0	0	0	0	0	0	0	0	0	0	0	0	-1	0	0	0	0	0
G3 →	1	0	0	1	0	0	0	0	0	0	0	0	0	0	0	1	0	0	1	0
G4 →	0	0	0	0	-1	0	0	0	0	0	0	0	0	0	0	0	0	0	0	0
G5 →	0	0	0	0	0	0	0	0	0	0	0	0	0	0	0	0	0	0	0	1
G6 →	0	0	1	0	0	0	-1	-1	1	-1	1	-1	1	1	0	0	1	0	0	0
G7 →	0	0	0	0	0	0	0	0	0	0	0	0	0	0	0	0	0	0	0	0
G8 →	0	0	0	0	0	0	0	0	0	0	0	0	0	0	0	0	0	0	0	0
G9 →	0	0	0	0	0	0	0	0	0	0	0	0	0	0	0	0	0	0	0	0
G10 →	0	0	0	0	0	0	0	0	0	0	0	0	0	0	0	0	0	-1	0	0
G11 →	0	0	0	0	0	0	0	0	0	0	0	0	0	0	0	0	0	-1	0	0
G12 →	0	0	0	0	0	0	0	0	0	0	0	0	0	0	0	0	0	0	0	0
G13 →	0	0	0	0	0	0	0	0	0	0	0	0	0	0	0	0	0	0	0	0
G14 →	0	0	0	0	0	0	0	0	0	0	0	0	0	0	0	0	0	0	0	0
G15 →	0	0	0	0	0	0	0	0	0	0	0	0	0	0	0	0	0	0	0	0
G16 →	0	0	0	0	0	0	0	0	0	0	0	0	0	0	0	0	0	0	0	0
G17 →	0	0	0	0	0	0	0	0	0	0	0	0	0	0	0	0	0	0	0	0
G18 →	0	0	0	0	0	0	0	0	0	0	0	0	0	0	0	0	0	0	0	0
G19 →	0	0	0	0	0	0	0	0	0	0	0	0	0	0	0	0	0	0	0	0
G20 →	0	0	0	0	0	0	0	0	0	0	0	0	0	0	0	0	0	0	0	0

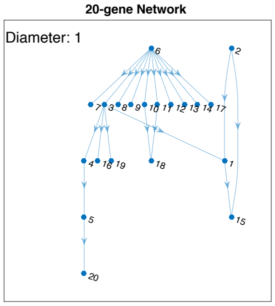

Network E. coli 10	G1	G2	G3	G4	G5	G6	G7	G8	G9	G10	G11	G12	G13	G14	G15	G16	G17	G18	G19	G20
G1 →	0	0	0	0	0	0	0	0	0	0	0	0	0	0	0	0	1	0	0	0
G2 →	1	0	0	0	0	0	0	0	0	0	0	0	0	0	0	0	1	0	0	0
G3 →	1	0	0	1	1	1	-1	1	-1	0	0	0	0	1	1	-1	1	1	1	1
G4 →	0	0	0	0	0	-1	0	0	0	0	0	0	0	0	0	0	0	0	0	0
G5 →	0	0	0	0	0	0	0	0	0	0	0	0	0	0	0	0	0	0	0	0
G6 →	0	0	0	-1	0	0	0	0	0	0	0	0	0	0	0	0	0	0	0	0
G7 →	0	0	0	0	0	0	0	0	0	0	0	0	0	0	0	0	0	0	0	-1
G8 →	0	0	0	0	0	0	0	0	0	0	1	0	1	1	1	0	0	1	1	0
G9 →	0	0	0	0	0	0	0	0	0	-1	0	1	0	0	0	1	0	0	0	0
G10 →	0	0	0	0	0	0	0	0	0	0	0	0	0	0	0	0	0	0	0	0
G11 →	0	0	0	0	0	0	0	0	0	0	0	0	1	0	0	0	0	0	0	0
G12 →	0	0	0	0	0	0	0	0	0	0	0	0	0	0	0	0	0	0	0	0
G13 →	0	0	0	0	0	0	0	0	0	0	0	0	0	0	0	0	0	0	0	0
G14 →	0	0	0	0	0	0	0	0	0	0	0	0	0	0	0	0	0	0	0	0
G15 →	0	0	0	0	0	0	0	0	0	0	0	0	0	0	0	0	0	0	0	0
G16 →	0	0	0	0	0	0	0	0	0	0	0	0	0	0	0	0	0	0	0	0
G17 →	0	0	0	0	0	0	0	0	0	0	0	0	0	0	0	0	0	0	0	0
G18 →	0	0	0	0	0	0	0	0	0	0	0	0	0	0	0	0	0	0	0	0
G19 →	0	0	0	0	0	0	0	0	0	0	0	0	0	0	0	0	0	0	0	0
G20 →	0	0	0	0	0	0	0	0	0	0	0	0	0	0	0	0	0	0	0	0

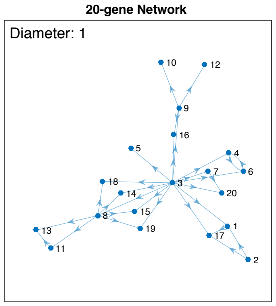

Network Yeast 1	G1	G2	G3	G4	G5	G6	G7	G8	G9	G10	G11	G12	G13	G14	G15	G16	G17	G18	G19	G20
G1 →	0	1	0	0	0	0	0	0	1	0	0	0	0	0	0	0	0	0	1	0
G2 →	0	0	0	0	0	0	0	0	0	0	1	1	0	1	0	1	0	0	0	0
G3 →	0	1	0	1	0	0	0	1	0	1	0	0	1	0	0	1	1	0	0	1
G4 →	0	0	0	0	0	0	0	0	0	0	0	0	1	0	0	0	0	1	0	0
G5 →	0	1	0	0	0	0	0	0	1	0	0	0	0	0	0	0	0	0	1	1
G6 →	0	1	0	0	0	0	1	0	0	0	0	1	0	1	0	0	0	0	0	0
G7 →	0	0	0	0	0	0	0	0	0	0	0	0	0	0	1	0	0	0	0	0
G8 →	0	0	0	0	0	0	0	0	0	0	0	0	0	0	0	0	0	0	0	0
G9 →	0	0	0	0	0	0	0	0	0	0	0	0	0	0	0	0	0	0	0	0
G10 →	0	0	0	0	0	0	0	0	0	0	0	0	0	0	0	0	0	0	0	0
G11 →	0	0	0	0	0	0	0	0	0	0	0	0	0	0	0	0	0	0	0	0
G12 →	0	0	0	0	0	0	0	0	0	0	0	0	0	0	0	0	0	0	0	0
G13 →	0	0	0	0	0	0	0	0	0	0	0	0	0	0	0	0	0	0	0	0
G14 →	0	0	0	0	0	0	0	0	0	0	0	0	0	0	0	0	0	0	0	0
G15 →	0	0	0	0	0	0	0	0	0	0	0	0	0	0	0	0	0	0	0	0
G16 →	0	0	0	0	0	0	0	0	0	0	0	0	0	0	0	0	0	0	0	0
G17 →	0	0	0	0	0	0	0	0	0	0	0	0	0	0	0	0	0	0	0	0
G18 →	0	0	0	0	0	0	0	0	0	0	0	0	0	0	0	0	0	0	0	0
G19 →	0	0	0	0	0	0	0	0	0	0	0	0	0	0	0	0	0	0	0	0
G20 →	0	0	0	0	0	0	0	0	0	0	0	0	0	0	0	0	0	0	0	0

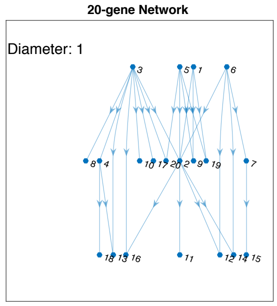

Network Yeast 2	G1	G2	G3	G4	G5	G6	G7	G8	G9	G10	G11	G12	G13	G14	G15	G16	G17	G18	G19	G20
G1 →	0	1	0	0	0	0	0	0	1	0	0	0	0	0	1	0	1	0	0	0
G2 →	0	0	0	0	0	0	1	1	0	1	0	1	0	1	0	0	0	1	0	1
G3 →	0	1	0	0	1	0	0	0	1	1	1	0	1	0	0	1	0	0	0	0
G4 →	0	1	0	0	0	0	0	0	0	0	0	0	0	0	1	0	1	0	0	0
G5 →	0	0	0	0	0	0	1	0	0	0	0	0	0	0	0	1	0	0	0	0
G6 →	0	1	0	0	0	0	0	1	0	0	0	1	0	1	0	0	0	1	1	1
G7 →	0	0	0	0	0	0	0	1	0	0	0	1	0	1	0	0	0	1	0	1
G8 →	0	0	0	0	0	0	0	0	0	0	0	0	0	0	0	0	0	0	0	0
G9 →	0	0	0	0	0	0	0	0	0	0	0	0	0	0	0	0	0	0	0	0
G10 →	0	0	0	0	0	0	0	0	0	0	0	0	0	0	0	0	0	0	0	0
G11 →	0	0	0	0	0	0	0	0	0	0	0	0	0	0	0	0	0	0	0	0
G12 →	0	0	0	0	0	0	0	0	0	0	0	0	0	0	0	0	0	0	0	0
G13 →	0	0	0	0	0	0	0	0	0	0	0	0	0	0	0	0	0	0	0	0
G14 →	0	0	0	0	0	0	0	0	0	0	0	0	0	0	0	0	0	0	0	0
G15 →	0	0	0	0	0	0	0	0	0	0	0	0	0	0	0	0	0	0	0	0
G16 →	0	0	0	0	0	0	0	0	0	0	0	0	0	0	0	0	0	0	0	0
G17 →	0	0	0	0	0	0	0	0	0	0	0	0	0	0	0	0	0	0	0	0
G18 →	0	0	0	0	0	0	0	0	0	0	0	0	0	0	0	0	0	0	0	0
G19 →	0	0	0	0	0	0	0	0	0	0	0	0	0	0	0	0	0	0	0	0
G20 →	0	0	0	0	0	0	0	0	0	0	0	0	0	0	0	0	0	0	0	0

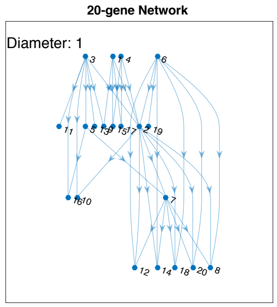

Network Yeast 3	G1	G2	G3	G4	G5	G6	G7	G8	G9	G10	G11	G12	G13	G14	G15	G16	G17	G18	G19	G20
G1 →	0	0	0	0	0	0	0	0	0	0	0	0	0	0	0	0	0	0	0	0
G2 →	1	0	0	0	0	0	0	0	1	0	0	0	0	1	0	0	0	0	0	0
G3 →	0	0	0	0	0	0	0	0	0	0	0	0	0	0	0	0	0	0	0	0
G4 →	0	0	1	0	1	1	1	1	0	1	1	1	1	0	1	1	1	1	1	1
G5 →	0	0	0	0	0	0	0	0	0	0	0	0	0	0	0	0	0	0	0	0
G6 →	0	0	0	0	0	0	0	0	0	0	0	0	0	0	0	0	0	0	0	0
G7 →	0	0	0	0	0	0	0	0	0	0	0	0	0	1	0	0	0	0	0	0
G8 →	0	0	0	0	0	0	0	0	0	0	0	0	0	0	0	0	0	0	0	0
G9 →	0	0	0	0	0	0	0	0	0	0	0	0	0	0	0	0	0	0	0	0
G10 →	0	0	0	0	0	0	0	0	0	0	0	0	0	0	0	0	0	0	0	0
G11 →	0	0	0	0	0	0	0	0	0	0	0	0	0	0	0	0	0	0	0	0
G12 →	0	0	0	0	0	0	0	0	0	0	0	0	0	0	0	0	0	0	0	0
G13 →	0	0	0	0	0	0	0	0	0	0	0	0	0	0	0	0	0	0	0	0
G14 →	0	0	0	0	0	0	0	0	0	0	0	0	0	0	0	0	0	0	0	0
G15 →	0	0	0	0	0	0	0	0	0	0	0	0	0	0	0	0	0	0	0	0
G16 →	0	0	0	0	0	0	0	0	0	0	0	0	0	0	0	0	0	0	0	0
G17 →	0	0	0	0	0	0	0	0	0	0	0	0	0	0	0	0	0	0	0	0
G18 →	0	0	0	0	0	0	0	0	0	0	0	0	0	0	0	0	0	0	0	0
G19 →	0	0	0	0	0	0	0	0	0	0	0	0	0	0	0	0	0	0	0	0
G20 →	0	0	0	0	0	0	0	0	0	0	0	0	0	0	0	0	0	0	0	0

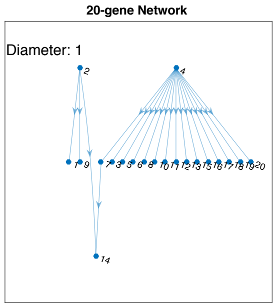

Network Yeast 4	G1	G2	G3	G4	G5	G6	G7	G8	G9	G10	G11	G12	G13	G14	G15	G16	G17	G18	G19	G20
G1 →	0	1	1	1	1	0	1	0	0	1	1	1	0	0	1	1	0	0	1	0
G2 →	0	0	0	0	0	0	0	1	0	0	0	0	1	1	0	0	0	0	0	0
G3 →	0	0	0	0	0	0	0	0	1	0	0	0	0	0	0	0	0	0	0	1
G4 →	0	0	0	0	0	0	0	1	0	0	0	0	1	1	0	0	1	1	0	0
G5 →	0	0	0	0	0	0	0	0	0	0	0	0	0	0	0	1	0	0	0	0
G6 →	0	0	0	1	0	0	0	0	0	0	0	0	0	0	0	0	0	0	0	0
G7 →	0	0	0	0	0	0	0	0	0	0	0	0	1	1	0	0	1	1	0	0
G8 →	0	0	0	0	0	0	0	0	0	0	0	0	0	0	0	0	0	0	0	0
G9 →	0	0	0	0	0	0	0	0	0	0	0	0	0	0	0	0	0	0	0	0
G10 →	0	0	0	0	0	0	0	0	0	0	0	0	0	0	0	0	0	0	0	0
G11 →	0	0	0	0	0	0	0	0	0	0	0	0	0	0	0	0	0	0	0	0
G12 →	0	0	0	0	0	0	0	0	0	0	0	0	0	0	0	0	0	0	0	0
G13 →	0	0	0	0	0	0	0	0	0	0	0	0	0	0	0	0	0	0	0	0
G14 →	0	0	0	0	0	0	0	0	0	0	0	0	0	0	0	0	0	0	0	0
G15 →	0	0	0	0	0	0	0	0	0	0	0	0	0	0	0	0	0	0	0	0
G16 →	0	0	0	0	0	0	0	0	0	0	0	0	0	0	0	0	0	0	0	0
G17 →	0	0	0	0	0	0	0	0	0	0	0	0	0	0	0	0	0	0	0	0
G18 →	0	0	0	0	0	0	0	0	0	0	0	0	0	0	0	0	0	0	0	0
G19 →	0	0	0	0	0	0	0	0	0	0	0	0	0	0	0	0	0	0	0	0
G20 →	0	0	0	0	0	0	0	0	0	0	0	0	0	0	0	0	0	0	0	0

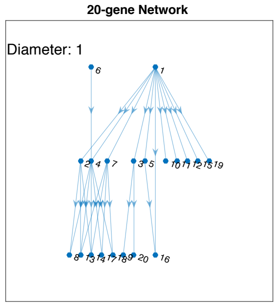

Network Yeast 5	G1	G2	G3	G4	G5	G6	G7	G8	G9	G10	G11	G12	G13	G14	G15	G16	G17	G18	G19	G20
G1 →	0	1	0	0	0	0	0	0	0	0	0	0	0	0	0	0	0	0	0	0
G2 →	0	0	0	0	0	0	0	0	0	0	0	0	0	0	0	0	1	0	0	0
G3 →	1	0	0	1	1	0	1	0	0	1	1	0	0	0	1	1	1	0	0	1
G4 →	1	0	0	0	1	0	1	0	0	1	1	0	0	0	1	1	0	0	0	1
G5 →	0	0	0	0	0	1	0	1	1	0	0	1	1	0	0	0	0	0	1	0
G6 →	0	0	0	0	0	0	0	0	0	0	0	0	0	0	0	0	0	0	0	0
G7 →	0	0	0	0	0	1	0	0	0	0	0	1	1	1	0	0	0	1	0	0
G8 →	0	0	0	0	0	0	0	0	0	0	0	0	0	0	0	0	0	0	0	0
G9 →	0	0	0	0	0	0	0	0	0	0	0	0	0	0	0	0	0	0	0	0
G10 →	0	0	0	0	0	0	0	0	0	0	0	0	0	0	0	0	0	0	0	0
G11 →	0	0	0	0	0	0	0	0	0	0	0	0	0	0	0	0	0	0	0	0
G12 →	0	0	0	0	0	0	0	0	0	0	0	0	0	0	0	0	0	0	0	0
G13 →	0	0	0	0	0	0	0	0	0	0	0	0	0	0	0	0	0	0	0	0
G14 →	0	0	0	0	0	0	0	0	0	0	0	0	0	0	0	0	0	0	0	0
G15 →	0	0	0	0	0	0	0	0	0	0	0	0	0	0	0	0	0	0	0	0
G16 →	0	0	0	0	0	0	0	0	0	0	0	0	0	0	0	0	0	0	0	0
G17 →	0	0	0	0	0	0	0	0	0	0	0	0	0	0	0	0	0	0	0	0
G18 →	0	0	0	0	0	0	0	0	0	0	0	0	0	0	0	0	0	0	0	0
G19 →	0	0	0	0	0	0	0	0	0	0	0	0	0	0	0	0	0	0	0	0
G20 →	0	0	0	0	0	0	0	0	0	0	0	0	0	0	0	0	0	0	0	0

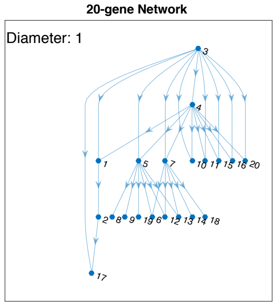

Network Yeast 6	G1	G2	G3	G4	G5	G6	G7	G8	G9	G10	G11	G12	G13	G14	G15	G16	G17	G18	G19	G20
G1 →	0	1	1	0	1	0	0	0	0	1	0	0	0	0	1	0	0	0	0	0
G2 →	0	0	0	0	0	0	0	0	0	0	0	0	0	0	0	0	0	0	0	0
G3 →	0	0	0	0	0	0	0	0	0	0	1	0	0	0	0	1	0	1	0	1
G4 →	0	0	1	0	0	0	0	0	0	0	0	0	1	0	0	0	0	0	0	0
G5 →	0	0	0	0	0	0	1	1	0	0	0	1	0	0	0	0	0	0	1	0
G6 →	0	0	1	0	0	0	0	0	1	0	0	0	0	1	0	0	0	0	0	1
G7 →	0	0	0	0	0	0	0	1	0	0	0	1	1	0	1	0	0	0	0	0
G8 →	0	0	0	0	0	0	0	0	0	0	0	0	0	0	0	0	0	0	0	0
G9 →	0	0	0	0	0	0	0	0	0	0	0	0	0	0	0	0	0	0	0	0
G10 →	0	0	0	0	0	0	0	0	0	0	0	0	0	0	0	0	0	0	0	0
G11 →	0	0	0	0	0	0	0	0	0	0	0	0	0	0	0	0	0	0	0	0
G12 →	0	0	0	0	0	0	0	0	0	0	0	0	0	0	0	0	0	0	0	0
G13 →	0	0	0	0	0	0	0	0	0	0	0	0	0	0	0	0	0	0	0	0
G14 →	0	0	0	0	0	0	0	0	0	0	0	0	0	0	0	0	0	0	0	0
G15 →	0	0	0	0	0	0	0	0	0	0	0	0	0	0	0	0	0	0	0	0
G16 →	0	0	0	0	0	0	0	0	0	0	0	0	0	0	0	0	0	0	0	0
G17 →	0	0	0	0	0	0	0	0	0	0	0	0	0	0	0	0	0	0	0	0
G18 →	0	0	0	0	0	0	0	0	0	0	0	0	0	0	0	0	0	0	0	0
G19 →	0	0	0	0	0	0	0	0	0	0	0	0	0	0	0	0	0	0	0	0
G20 →	0	0	0	0	0	0	0	0	0	0	0	0	0	0	0	0	0	0	0	0

20-gene Network

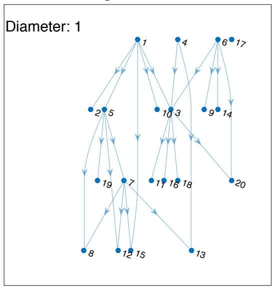

Network Yeast 7	G1	G2	G3	G4	G5	G6	G7	G8	G9	G10	G11	G12	G13	G14	G15	G16	G17	G18	G19	G20
G1 →	0	1	1	0	1	0	1	1	1	1	1	0	1	1	1	1	1	0	0	1
G2 →	0	0	0	0	0	0	0	0	0	0	0	0	0	0	0	0	0	0	1	0
G3 →	0	0	0	0	0	0	0	0	0	0	0	0	0	0	0	0	0	0	0	0
G4 →	0	0	1	0	0	1	0	0	0	0	0	0	0	0	0	0	0	0	0	0
G5 →	0	0	0	0	0	0	0	0	0	0	0	0	0	0	0	0	0	1	0	0
G6 →	0	0	0	0	0	0	0	0	0	0	0	0	0	0	0	0	0	0	0	0
G7 →	0	0	0	0	0	0	0	0	0	0	0	0	0	0	0	0	0	0	0	0
G8 →	0	0	0	0	0	0	0	0	0	0	0	0	0	0	0	0	0	0	0	0
G9 →	0	0	0	0	0	0	0	0	0	0	0	0	0	0	0	0	0	0	0	0
G10 →	0	0	0	0	0	0	0	0	0	0	0	0	0	0	0	0	0	0	0	0
G11 →	0	0	0	0	0	0	0	0	0	0	0	0	0	0	0	0	0	0	0	0
G12 →	0	0	0	0	0	0	0	0	0	0	0	0	0	0	0	0	0	0	0	0
G13 →	0	0	0	0	0	0	0	0	0	0	0	0	0	0	0	0	0	0	0	0
G14 →	0	0	0	0	0	0	0	0	0	0	0	0	0	0	0	0	0	0	0	0
G15 →	0	0	0	0	0	0	0	0	0	0	0	0	0	0	0	0	0	0	0	0
G16 →	0	0	0	0	0	0	0	0	0	0	0	0	0	0	0	0	0	0	0	0
G17 →	0	0	0	0	0	0	0	0	0	0	0	0	0	0	0	0	0	0	0	0
G18 →	0	0	0	0	0	0	0	0	0	0	0	0	0	0	0	0	0	0	0	0
G19 →	0	0	0	0	0	0	0	0	0	0	0	0	0	0	0	0	0	0	0	0
G20 →	0	0	0	0	0	0	0	0	0	0	0	0	0	0	0	0	0	0	0	0

20-gene Network

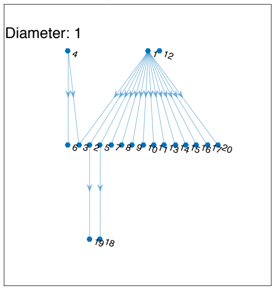

Network Yeast 8	G1	G2	G3	G4	G5	G6	G7	G8	G9	G10	G11	G12	G13	G14	G15	G16	G17	G18	G19	G20
G1 →	0	1	0	0	0	0	0	0	0	0	0	0	0	0	1	0	0	0	0	0
G2 →	0	0	0	0	0	0	0	0	0	0	0	0	0	0	1	0	0	0	0	0
G3 →	1	1	0	0	1	0	0	1	0	0	1	0	0	1	0	0	0	1	0	1
G4 →	1	1	0	0	0	0	0	0	1	1	0	0	0	0	0	0	1	0	0	0
G5 →	0	1	1	0	0	1	0	1	0	0	1	1	1	0	0	1	1	1	1	1
G6 →	1	0	1	0	0	0	0	0	1	1	0	1	1	1	0	0	0	0	1	0
G7 →	0	1	0	0	0	0	0	0	0	0	0	0	0	0	1	0	0	0	0	0
G8 →	0	0	0	0	0	0	0	0	0	0	0	0	0	0	0	0	0	0	0	0
G9 →	0	0	0	0	0	0	0	0	0	0	0	0	0	0	0	0	0	0	0	0
G10 →	0	0	0	0	0	0	0	0	0	0	0	0	0	0	0	0	0	0	0	0
G11 →	0	0	0	0	0	0	0	0	0	0	0	0	0	0	0	0	0	0	0	0
G12 →	0	0	0	0	0	0	0	0	0	0	0	0	0	0	0	0	0	0	0	0
G13 →	0	0	0	0	0	0	0	0	0	0	0	0	0	0	0	0	0	0	0	0
G14 →	0	0	0	0	0	0	0	0	0	0	0	0	0	0	0	0	0	0	0	0
G15 →	0	0	0	0	0	0	0	0	0	0	0	0	0	0	0	0	0	0	0	0
G16 →	0	0	0	0	0	0	0	0	0	0	0	0	0	0	0	0	0	0	0	0
G17 →	0	0	0	0	0	0	0	0	0	0	0	0	0	0	0	0	0	0	0	0
G18 →	0	0	0	0	0	0	0	0	0	0	0	0	0	0	0	0	0	0	0	0
G19 →	0	0	0	0	0	0	0	0	0	0	0	0	0	0	0	0	0	0	0	0
G20 →	0	0	0	0	0	0	0	0	0	0	0	0	0	0	0	0	0	0	0	0

20-gene Network

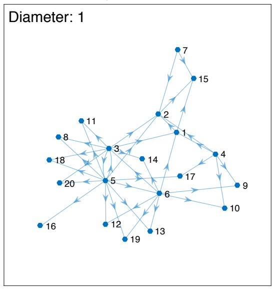

Network Yeast 9	G1	G2	G3	G4	G5	G6	G7	G8	G9	G10	G11	G12	G13	G14	G15	G16	G17	G18	G19	G20
G1 →	0	1	0	0	1	0	0	0	0	1	0	0	1	0	1	1	0	1	1	1
G2 →	0	0	0	0	0	0	0	0	0	0	0	0	0	0	0	0	1	0	0	0
G3 →	0	1	0	0	0	0	0	0	0	0	0	1	0	0	0	0	1	1	0	0
G4 →	0	1	0	0	0	0	0	1	0	0	0	1	0	0	0	0	0	1	0	0
G5 →	1	1	0	0	0	1	0	0	1	0	1	0	1	1	1	0	0	1	0	0
G6 →	0	1	0	0	0	0	0	0	0	0	0	0	0	0	0	0	1	1	0	0
G7 →	0	0	1	0	0	1	0	0	0	0	0	0	0	0	0	0	0	0	0	0
G8 →	0	0	0	0	0	0	0	0	0	0	0	0	0	0	0	0	0	0	0	0
G9 →	0	0	0	0	0	0	0	0	0	0	0	0	0	0	0	0	0	0	0	0
G10 →	0	0	0	0	0	0	0	0	0	0	0	0	0	0	0	0	0	0	0	0
G11 →	0	0	0	0	0	0	0	0	0	0	0	0	0	0	0	0	0	0	0	0
G12 →	0	0	0	0	0	0	0	0	0	0	0	0	0	0	0	0	0	0	0	0
G13 →	0	0	0	0	0	0	0	0	0	0	0	0	0	0	0	0	0	0	0	0
G14 →	0	0	0	0	0	0	0	0	0	0	0	0	0	0	0	0	0	0	0	0
G15 →	0	0	0	0	0	0	0	0	0	0	0	0	0	0	0	0	0	0	0	0
G16 →	0	0	0	0	0	0	0	0	0	0	0	0	0	0	0	0	0	0	0	0
G17 →	0	0	0	0	0	0	0	0	0	0	0	0	0	0	0	0	0	0	0	0
G18 →	0	0	0	0	0	0	0	0	0	0	0	0	0	0	0	0	0	0	0	0
G19 →	0	0	0	0	0	0	0	0	0	0	0	0	0	0	0	0	0	0	0	0
G20 →	0	0	0	0	0	0	0	0	0	0	0	0	0	0	0	0	0	0	0	0

20-gene Network

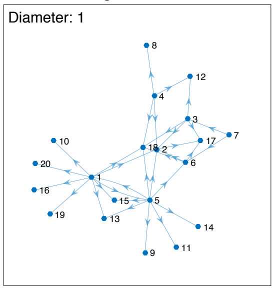

Network Yeast 10	G1	G2	G3	G4	G5	G6	G7	G8	G9	G10	G11	G12	G13	G14	G15	G16	G17	G18	G19	G20
G1 →	0	1	1	1	0	0	0	1	1	0	1	1	0	1	1	1	1	1	0	1
G2 →	0	0	0	0	0	0	0	0	0	0	0	0	0	0	0	0	0	0	0	0
G3 →	0	0	0	0	0	0	0	0	0	0	0	0	0	0	0	0	0	0	0	0
G4 →	0	0	0	0	0	0	0	0	0	0	0	0	0	0	0	0	0	0	0	0
G5 →	0	1	1	1	0	1	0	0	1	0	1	0	1	0	1	1	0	0	1	1
G6 →	0	0	0	1	0	0	1	0	0	1	0	1	1	1	1	0	1	0	1	0
G7 →	0	0	0	0	1	1	0	0	0	0	0	0	0	1	1	0	1	0	0	1
G8 →	0	0	0	0	0	0	0	0	0	0	0	0	0	0	0	0	0	0	0	0
G9 →	0	0	0	0	0	0	0	0	0	0	0	0	0	0	0	0	0	0	0	0
G10 →	0	0	0	0	0	0	0	0	0	0	0	0	0	0	0	0	0	0	0	0
G11 →	0	0	0	0	0	0	0	0	0	0	0	0	0	0	0	0	0	0	0	0
G12 →	0	0	0	0	0	0	0	0	0	0	0	0	0	0	0	0	0	0	0	0
G13 →	0	0	0	0	0	0	0	0	0	0	0	0	0	0	0	0	0	0	0	0
G14 →	0	0	0	0	0	0	0	0	0	0	0	0	0	0	0	0	0	0	0	0
G15 →	0	0	0	0	0	0	0	0	0	0	0	0	0	0	0	0	0	0	0	0
G16 →	0	0	0	0	0	0	0	0	0	0	0	0	0	0	0	0	0	0	0	0
G17 →	0	0	0	0	0	0	0	0	0	0	0	0	0	0	0	0	0	0	0	0
G18 →	0	0	0	0	0	0	0	0	0	0	0	0	0	0	0	0	0	0	0	0
G19 →	0	0	0	0	0	0	0	0	0	0	0	0	0	0	0	0	0	0	0	0
G20 →	0	0	0	0	0	0	0	0	0	0	0	0	0	0	0	0	0	0	0	0

20-gene Network

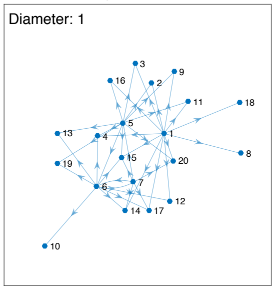

Supplement: Supplementary Data [file btx575_supp.zip › btx575-suppl_data/Supplementary File.pdf]
