# Supplementary material for: SINCERITIES: inferring gene regulatory networks from time-stamped single cell transcriptional expression profiles
Source: Bioinformatics. 2017 Sep 14;34(2):258–66. doi: 10.1093/bioinformatics/btx575 (PMC5860204; doi:10.1093/bioinformatics/btx575)
Supplement: Supplementary Data [file btx575_supp.zip › btx575-suppl_data/Supplementary Material SINCERITIES.pdf]

Supplementary Material for

SINCERITIES: Inferring gene regulatory network from time-stamped cross-sectional single cell transcriptional expression data

Nan Papili Gao[1,2], S.M. Minhaz Ud-Dean[3], Olivier Gandrillon[4,5], and Rudiyanto Gunawan[1,2,*]

[1]Institute for Chemical and Bioengineering, ETH Zurich, Zurich, Switzerland

[2]Swiss Institute of Bioinformatics, Lausanne, Switzerland

[3]Department of Environmental Health Sciences, Mailman School of Public Health, Columbia University, New York, NY, USA.

[4]Univ Lyon, ENS de Lyon, Univ Claude Bernard, CNRS UMR 5239, INSERM U1210, Laboratory of Biology and Modelling of the Cell, 46 allée d'Italie Site Jacques Monod, F-69007, Lyon, France

[5]Inria Team Dracula, Inria Center Grenoble Rhône-Alpes, France

Alternative distribution distances

We evaluated the use of the Cramér–von Mises (CM) criterion (Anderson, 1962) as the DD metric. The CM criterion is given by:

$$CM_{j,\Delta t_l} = \int_{-\infty}^{\infty} \left(F_{t_{l+1}}(E_j) - F_{t_l}(E_j)\right)^2 dF_{t_l}(E_j) \qquad \text{(S1)}$$

where $CM_{j,\Delta t_l}$ denotes the CM criterion of gene j in the time window Δt_l, and $F_{t_l}(E_j)$ denotes the cumulative distribution function of gene j expression (E_j) at time point t_l (l = 1, 2, …, n−1). In addition to the CM criterion, we tested the Anderson-Darling (AD) criterion (Anderson and Darling, 1952), which is given by:

$$AD_{j,\Delta t_l} = \int_{-\infty}^{\infty} \frac{\left(F_{t_{l+1}}(E_j) - F_{t_l}(E_j)\right)^2}{F_{t_l}(E_j)\left(1 - F_{t_l}(E_j)\right)} dF_{t_l}(E_j) \qquad \text{(S2)}$$

The CM and AD criteria provide more sensitive measures of the global change in the distribution than the KS distance (Stephens, 1970). In contrast, the KS distance better reflects the shift of the center of the distribution. Finally, we also applied SINCERITIES using mean difference as the DD metric. Table S1 reports the AUROCs and AUPRs of SINCERITIES using the KS, mean difference, CM and AD criteria. The AUROC and AUPR values showed that other DD metrics could provide a comparable performance to the KS distance. However, for the THP-1 differentiation dataset, mean difference, CM and AD distances gave poorer AUROC and AUPR values than the KS distance (AUROC: 0.52 for mean, 0.54 for CM, 0.56 for AD vs. 0.70 for KS, AUPR: 0.21 for mean, 0.20 for CM, 0.22 for AD vs. 0.33 for KS).

Table S1. Performance comparison: SINCERITIES using KS, mean, AD or CM distances on *in silico* datasets.

	**SINCERITIES**															
	**10-GENE NETWORK**								**20-GENE NETWORK**							
	**AUROC**				**AUPR**				**AUROC**				**AUPR**			
	KS	*CM*	*AD*	*MEAN*	*KS*	*CM*	*AD*	*MEAN*	*KS*	*CM*	*AD*	*MEAN*	*KS*	*CM*	*AD*	*MEAN*
Network E. coli 1	0.65	0.68	0.67	0.56	0.14	0.13	0.13	0.16	0.48	0.51	0.47	0.56	0.09	0.18	0.09	0.14
Network E. coli 2	0.71	0.78	0.78	0.58	0.15	0.19	0.19	0.10	0.44	0.42	0.42	0.37	0.06	0.05	0.05	0.04
Network E. coli 3	0.77	0.75	0.78	0.42	0.15	0.22	0.20	0.08	0.75	0.74	0.73	0.29	0.19	0.15	0.15	0.05
Network E. coli 4	0.85	0.87	0.85	0.46	0.36	0.37	0.34	0.14	0.57	0.57	0.57	0.35	0.08	0.08	0.08	0.05
Network E. coli 5	0.80	0.85	0.85	0.64	0.19	0.25	0.26	0.14	0.55	0.72	0.70	0.42	0.07	0.13	0.12	0.05
Network E. coli 6	0.59	0.69	0.64	0.50	0.12	0.16	0.14	0.11	0.81	0.89	0.89	0.67	0.27	0.39	0.37	0.09
Network E. coli 7	0.54	0.46	0.46	0.66	0.17	0.15	0.15	0.27	0.75	0.78	0.78	0.25	0.16	0.15	0.16	0.03
Network E. coli 8	0.83	0.80	0.80	0.26	0.23	0.20	0.20	0.06	0.82	0.91	0.91	0.74	0.28	0.45	0.46	0.09
Network E. coli 9	0.79	0.84	0.85	0.62	0.29	0.46	0.52	0.15	0.71	0.74	0.74	0.50	0.10	0.11	0.11	0.05
Network E. coli 10	0.88	0.90	0.90	0.23	0.35	0.32	0.32	0.06	0.58	0.59	0.60	0.61	0.07	0.08	0.08	0.08
Network Yeast 11	0.69	0.69	0.69	0.78	0.26	0.37	0.37	0.33	0.70	0.75	0.74	0.74	0.18	0.29	0.26	0.27
Network Yeast 12	0.64	0.72	0.74	0.89	0.13	0.25	0.25	0.45	0.73	0.73	0.73	0.75	0.27	0.30	0.28	0.32
Network Yeast 13	0.84	0.83	0.83	0.82	0.68	0.67	0.65	0.59	0.60	0.73	0.73	0.93	0.06	0.08	0.08	0.29
Network Yeast 14	0.84	0.85	0.85	0.90	0.57	0.57	0.57	0.80	0.63	0.71	0.71	0.78	0.09	0.17	0.14	0.14
Network Yeast 15	0.86	0.89	0.89	0.86	0.44	0.53	0.53	0.51	0.71	0.64	0.65	0.74	0.31	0.29	0.30	0.32
Network Yeast 16	0.90	0.89	0.90	0.91	0.48	0.50	0.52	0.59	0.70	0.72	0.72	0.73	0.17	0.18	0.17	0.23
Network Yeast 17	0.78	0.76	0.77	0.89	0.39	0.36	0.37	0.58	0.73	0.82	0.81	0.89	0.13	0.17	0.14	0.31
Network Yeast 18	0.92	0.93	0.94	0.95	0.72	0.73	0.78	0.79	0.65	0.69	0.70	0.73	0.17	0.18	0.18	0.21
Network Yeast 19	0.73	0.81	0.81	0.84	0.23	0.52	0.52	0.69	0.78	0.81	0.82	0.80	0.26	0.24	0.25	0.24
Network Yeast 20	0.94	0.93	0.94	0.95	0.73	0.69	0.72	0.84	0.73	0.81	0.81	0.83	0.20	0.33	0.36	0.39
**Mean**	**0.78**	**0.80**	**0.80**	**0.69**	**0.34**	**0.38**	**0.39**	**0.37**	**0.67**	**0.71**	**0.71**	**0.63**	**0.16**	**0.20**	**0.19**	**0.17**
**± SD**	**0.11**	**0.11**	**0.11**	**0.23**	**0.20**	**0.19**	**0.20**	**0.28**	**0.10**	**0.12**	**0.12**	**0.21**	**0.08**	**0.11**	**0.12**	**0.12**

Alternative regularization methods: Lasso and Elastic-net

While we recommended using ridge regression, SINCERITIES could also be implemented using two additional regularization strategies, namely Lasso (Least Absolute Shrinkage and Selection Operator) and elastic-net. The three methods differ only in the penalty function used in the least square objective function in Eq. (4) in the main text. In contrast to ridge regression, the Lasso regularization enforces an L1 norm penalty in the least square objective function, as follows

$$\min_{\boldsymbol{\alpha}} \|\mathbf{y} - \mathbf{X}\boldsymbol{\alpha}\|_2^2 + \lambda \, \|\boldsymbol{\alpha}\|_1 \tag{S3}$$

Meanwhile, the elastic net uses a penalty function that combines those from the Lasso and ridge regression, with the following least square objective function:

$$\min_{\boldsymbol{\alpha}} \|\mathbf{y} - \mathbf{X}\boldsymbol{\alpha}\|_2^2 + \lambda \left((1-\gamma)/2 \, \|\alpha\|_2^2 + \gamma \|\boldsymbol{\alpha}\|_1\right) \tag{S4}$$

Setting γ to 1 would give the Lasso regularization, while setting γ to 0 would give the ridge regression. Here, we again used LOOCV to determine the parameters λ and γ. In the case of elastic net, we performed LOOCV to obtain the optimal λ value for discrete values of γ between 0.1 and 0.9 with a step size of 0.1 (i.e. γ = 0.1, 0.2, …, 0.9). The final optimal combination of λ and γ again corresponded to the minimum cross validation error among the LOOCV runs.

Table S2 reports the performance of SINCERITIES using the KS distance using the Lasso and elastic net regularization strategies for the *in silico* single cell dataset. For 10-gene gold standard GRNs, the ridge regression gave significantly higher AUROCs and AUPRs (p-value<0.05, paired t-tests) than the Lasso and elastic net.

Table S2. Performance comparison: SINCERITIES using KS distance with Ridge, Elastic-net, and Lasso on *in silico* datasets.

	SINCERITIES											
	10-GENE NETWORK						20-GENE NETWORK					
	AUROC			AUPR			AUROC			AUPR		
	RIDGE	ELASTIC-NET	LASSO	RIDGE	ELASTIC-NET	LASSO	RIDGE	ELASTIC-NET	LASSO	RIDGE	ELASTIC-NET	LASSO
Network E. coli 1	0.65	0.50	0.52	0.14	0.10	0.11	0.48	0.46	0.49	0.09	0.09	0.08
Network E. coli 2	0.71	0.57	0.54	0.15	0.23	0.22	0.44	0.51	0.47	0.06	0.06	0.02
Network E. coli 3	0.77	0.63	0.55	0.15	0.12	0.10	0.75	0.44	0.48	0.19	0.07	0.04
Network E. coli 4	0.85	0.47	0.45	0.36	0.11	0.07	0.57	0.58	0.48	0.08	0.12	0.04
Network E. coli 5	0.80	0.67	0.61	0.19	0.17	0.19	0.55	0.43	0.47	0.07	0.05	0.02
Network E. coli 6	0.59	0.53	0.43	0.12	0.11	0.03	0.81	0.51	0.51	0.27	0.10	0.08
Network E. coli 7	0.54	0.52	0.56	0.17	0.19	0.28	0.75	0.47	0.48	0.16	0.04	0.03
Network E. coli 8	0.83	0.52	0.46	0.23	0.15	0.05	0.82	0.49	0.54	0.28	0.10	0.14
Network E. coli 9	0.79	0.62	0.58	0.29	0.21	0.20	0.71	0.62	0.54	0.10	0.10	0.08
Network E. coli 10	0.88	0.52	0.44	0.35	0.11	0.02	0.58	0.46	0.47	0.07	0.06	0.04
Network Yeast 11	0.69	0.66	0.58	0.26	0.33	0.28	0.70	0.59	0.57	0.18	0.09	0.12
Network Yeast 12	0.64	0.41	0.45	0.13	0.06	0.04	0.73	0.52	0.49	0.27	0.10	0.08
Network Yeast 13	0.84	0.45	0.49	0.68	0.17	0.18	0.60	0.49	0.46	0.06	0.04	0.02
Network Yeast 14	0.84	0.67	0.59	0.57	0.28	0.25	0.63	0.60	0.52	0.09	0.10	0.08
Network Yeast 15	0.86	0.52	0.47	0.44	0.12	0.09	0.71	0.54	0.53	0.31	0.16	0.15
Network Yeast 16	0.90	0.51	0.53	0.48	0.16	0.18	0.70	0.53	0.54	0.17	0.12	0.14
Network Yeast 17	0.78	0.81	0.75	0.39	0.45	0.46	0.73	0.48	0.47	0.13	0.04	0.02
Network Yeast 18	0.92	0.62	0.55	0.72	0.30	0.24	0.65	0.49	0.51	0.17	0.10	0.11
Network Yeast 19	0.73	0.52	0.48	0.23	0.12	0.10	0.78	0.61	0.52	0.26	0.13	0.10
Network Yeast 20	0.94	0.77	0.56	0.73	0.46	0.23	0.73	0.56	0.51	0.20	0.17	0.15
**Mean**	**0.78**	**0.57**	**0.53**	**0.34**	**0.20**	**0.17**	**0.67**	**0.52**	**0.50**	**0.16**	**0.09**	**0.08**
**± SD**	**0.11**	**0.11**	**0.08**	**0.20**	**0.11**	**0.11**	**0.10**	**0.06**	**0.03**	**0.08**	**0.04**	**0.05**

Table S3. Performance comparison among TSNI, GENIE3, JUMP3, and SINCERITIES on *in silico* datasets.

	10-GENE NETWORK								20-GENE NETWORK							
	AUROC				AUPR				AUROC				AUPR			
	TSNI	*GENIE3*	*JUMP3*	*SINCERITIES*	*TSNI*	*GENIE3*	*JUMP3*	*SINCERITIES*	*TSNI*	*GENIE3*	*JUMP3*	*SINCERITIES*	*TSNI*	*GENIE3*	*JUMP3*	*SINCERITIES*
Network E. coli 1	0.52	0.28	0.21	0.65	0.23	0.04	0.05	0.14	0.45	0.52	0.05	0.47	0.11	0.14	0.01	0.09
Network E. coli 2	0.40	0.27	0.26	0.71	0.07	0.04	0.05	0.15	0.42	0.57	0.04	0.44	0.05	0.05	0.00	0.06
Network E. coli 3	0.25	0.29	0.40	0.77	0.05	0.04	0.05	0.15	0.61	0.29	0.06	0.73	0.08	0.03	0.01	0.19
Network E. coli 4	0.31	0.30	0.15	0.85	0.19	0.07	0.04	0.36	0.56	0.45	0.04	0.55	0.08	0.04	0.01	0.08
Network E. coli 5	0.49	0.30	0.45	0.80	0.11	0.05	0.09	0.19	0.36	0.24	0.06	0.51	0.05	0.04	0.00	0.07
Network E. coli 6	0.34	0.45	0.17	0.59	0.18	0.07	0.03	0.12	0.40	0.28	0.08	0.79	0.13	0.03	0.02	0.27
Network E. coli 7	0.32	0.66	0.34	0.54	0.17	0.31	0.13	0.17	0.49	0.23	0.03	0.75	0.05	0.02	0.00	0.16
Network E. coli 8	0.45	0.22	0.23	0.83	0.06	0.03	0.03	0.23	0.35	0.14	0.12	0.81	0.13	0.03	0.03	0.28
Network E. coli 9	0.35	0.26	0.19	0.79	0.14	0.15	0.03	0.29	0.49	0.40	0.09	0.72	0.05	0.03	0.02	0.10
Network E. coli 10	0.30	0.17	0.12	0.88	0.09	0.02	0.02	0.35	0.46	0.36	0.06	0.58	0.07	0.04	0.05	0.07
Network Yeast 11	0.40	0.56	0.24	0.69	0.10	0.12	0.05	0.26	0.44	0.30	0.08	0.65	0.15	0.07	0.02	0.18
Network Yeast 12	0.32	0.27	0.45	0.64	0.06	0.05	0.21	0.13	0.49	0.38	0.05	0.69	0.20	0.18	0.02	0.27
Network Yeast 13	0.56	0.42	0.34	0.84	0.18	0.11	0.15	0.68	0.16	0.12	0.08	0.57	0.03	0.03	0.02	0.06
Network Yeast 14	0.37	0.24	0.33	0.84	0.13	0.09	0.20	0.57	0.43	0.44	0.09	0.60	0.07	0.07	0.06	0.09
Network Yeast 15	0.40	0.36	0.40	0.86	0.13	0.09	0.11	0.44	0.31	0.46	0.13	0.63	0.06	0.07	0.05	0.31
Network Yeast 16	0.44	0.23	0.37	0.90	0.10	0.07	0.17	0.48	0.52	0.43	0.10	0.62	0.07	0.10	0.06	0.17
Network Yeast 17	0.40	0.26	0.35	0.78	0.09	0.06	0.27	0.39	0.22	0.21	0.12	0.74	0.03	0.03	0.08	0.13
Network Yeast 18	0.32	0.22	0.32	0.92	0.16	0.16	0.11	0.72	0.42	0.34	0.10	0.60	0.12	0.07	0.07	0.17
Network Yeast 19	0.44	0.34	0.33	0.73	0.12	0.11	0.10	0.23	0.54	0.40	0.11	0.70	0.11	0.06	0.05	0.26
Network Yeast 20	0.31	0.18	0.39	0.94	0.12	0.09	0.17	0.73	0.35	0.21	0.08	0.68	0.08	0.06	0.02	0.20
**Mean**	**0.38**	**0.31**	**0.30**	**0.78**	**0.12**	**0.09**	**0.10**	**0.34**	**0.42**	**0.34**	**0.08**	**0.64**	**0.09**	**0.06**	**0.03**	**0.16**
**± SD**	**0.08**	**0.12**	**0.10**	**0.11**	**0.05**	**0.06**	**0.07**	**0.20**	**0.11**	**0.12**	**0.03**	**0.10**	**0.04**	**0.04**	**0.02**	**0.08**

Table S4. Up-, Mid- and Downstream Gene Lists. The gene names are listed in decreasing order of the ratio between out- and in-degree.

Upstream	Midstream	Downstream
NSDHL	ATXN2	TBC1D7
PTPRC	HRAS	HSD17B7
HYAL1	CREG1	SQSTM1
EMB	DCTD	FHL3
BATF	GAB1	DHCR7
TADA2A	CD151	RNASE2
SLC25A37	RPL22L1	SERPINI1
MID2	FAM208B	MFSD2B
ABCG2	SCD	ANGPTL4
AACS	DPP7	UCK1
PLS3	RUNX2	ACSL6
ACSS1	REXO2	AMDHD2
ALAS1	STX12	RFFL
SULF2	STARD4	PPP1R15B
LCP1	HMGCS1	LDHA
EGFR	HSP90AA1	PDLIM7
RBM38	VDAC3	SLC6A9
SQLE	GPT2	MKNK2
PIK3CG	MTFR1	NCOA4
PLAG1	SULT1E1	RHPN2
TPP1	MYO1G	SMPD1
XPNPEP1	PAPD5	HBB
SNX27	GSN	MAPK12
CTSA	CYP51A1	FNIP1
DHCR24	WDR91	CTCF
CD44	VRK3	GLRX5
BPI	TNFRSF21	TTYH2
BCL11A	PLS1	UNKNOWN8
MVD	SLC9A3R2	SNX22
ARHGEF2	CRIP2	
DCP1A		

Table S5. Gene Ontology Enrichment Analysis of Up-, Mid- and Downstream Genes in T2EC Differentiation based on top 500 edges of SINCERITIES.

Enriched GO Biological Process Terms	-log10p		
	Upstream	Midstream	Downstream
cholesterol biosynthetic process	10.0000*	-	-
secondary alcohol biosynthetic process	10.0000*	-	-
sterol biosynthetic process	10.0000*	-	-
cell activation	-	5.2518*	-
ERBB2 signaling pathway	-	-	-

(*) Bonferroni-corrected p-value<0.05

Supplementary figures

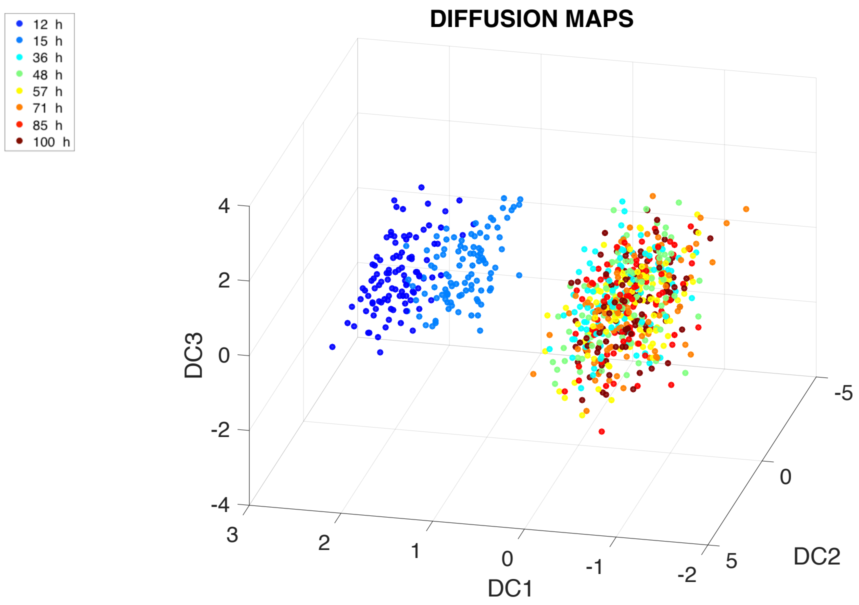

Fig. S1. Three-dimensional projection of *in silico* single cell data (10-gene Network *E. coli* 1) using diffusion map (Coifman and Lafon, 2006).

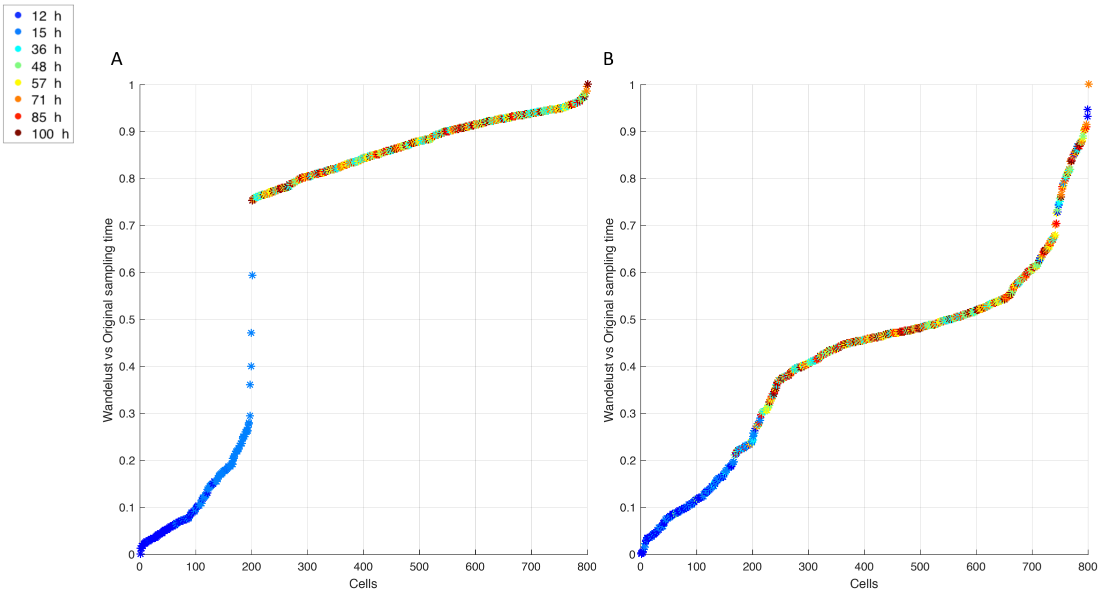

Fig. S2 Comparison between Wanderlust (Bendall et al., 2014) pseudotime and the true cell sampling time points for *in silico* single cell data (10-gene Network *E. coli* 1). Wanderlust algorithm was applied to (A) the original dataset in high-dimensional space, and (B) low-dimensional (3D) diffusion map projection data.

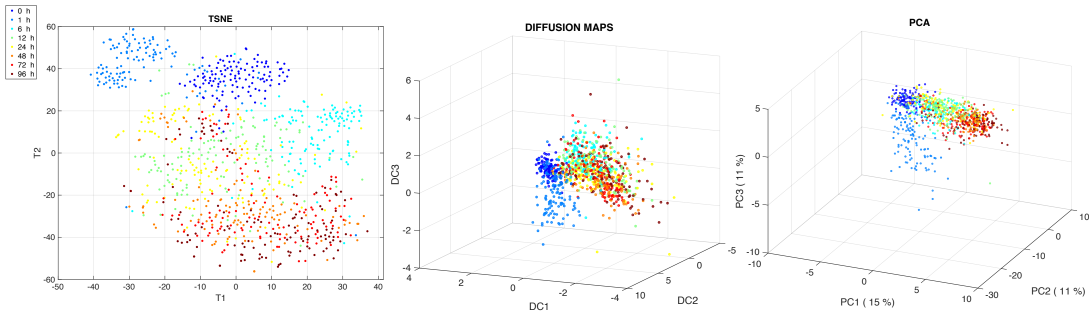

Fig. S3. Low dimensional projection of THP-1 human myeloid leukemia cell differentiation data using (a) principal component analysis (PCA), (b) t-Distributed Stochastic Neighbor Embedding (t-SNE) (Van Der Maaten and Hinton, 2008) and (c) diffusion map analysis.

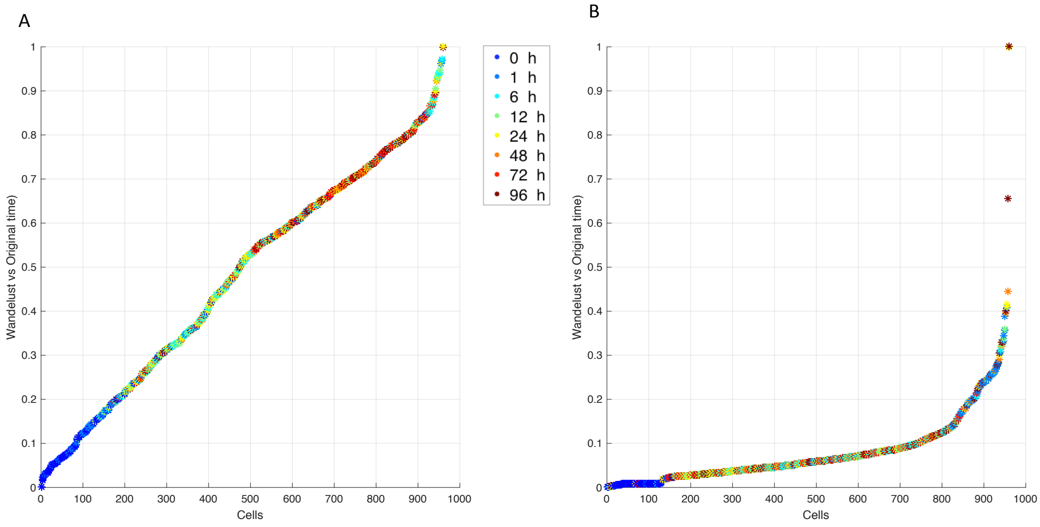

Fig. S4. Comparison between Wanderlust pseudotime and the true cell sampling time points of THP-1 human myeloid leukemia cell differentiation. Wanderlust algorithm was applied to (A) the original dataset in high-dimensional space, and (B) low-dimensional diffusion map projection data.

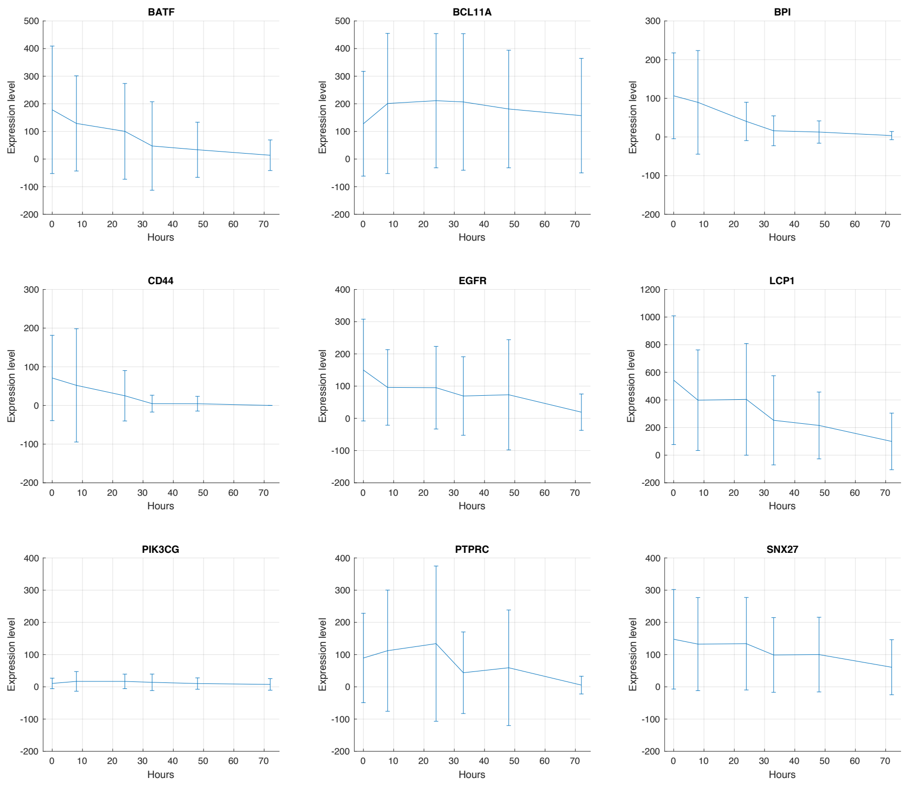

Fig. S5. Gene expression (mean ± SD) of 9 genes in T2EC dataset associated with the gene ontology biological process of cell activation. Overall, the expression of BATF, BPI, CD44, EGFR, LCP1, PTPRC and SNX27 were downregulated, while BCL11A was upregulated. The expression of PIK3CG and were invariant.

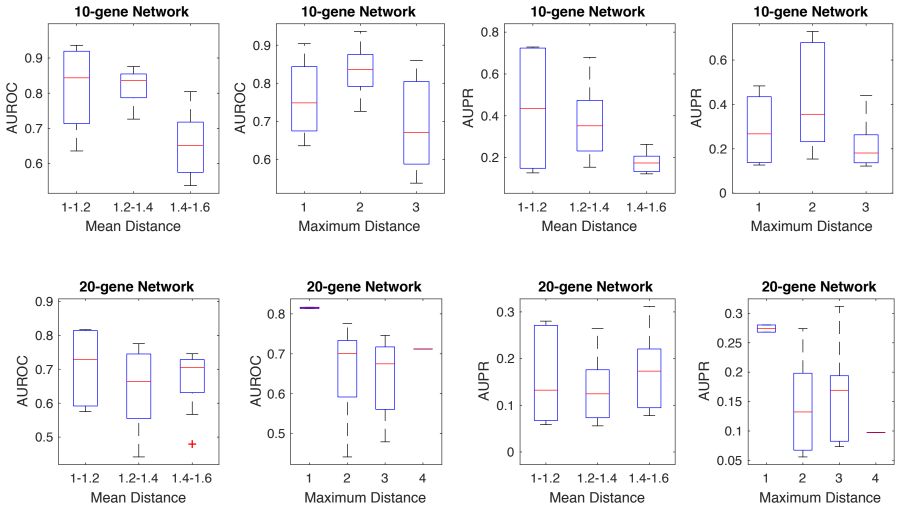

Fig. S6. AUROC and AUPR values of SINCERITIES using *in silico* single cell dataset: Effects of mean and maximum distances among the 10- and 20-gene *in silico* networks.

References

Anderson, T.W. (1962). On the Distribution of the Two-Sample Cramer-von Mises Criterion. Ann. Math. Stat. *33*, 1148–1159.

Anderson, T.W., and Darling, D.A. (1952). Asymptotic Theory of Certain "Goodness of Fit" Criteria Based on Stochastic Processes. Ann. Math. Stat. *23*, 193–212.

Bendall, S.C., Davis, K.L., Amir, E.-A.D., Tadmor, M.D., Simonds, E.F., Chen, T.J., Shenfeld, D.K., Nolan, G.P., and Pe'er, D. (2014). Single-cell trajectory detection uncovers progression and regulatory coordination in human B cell development. Cell *157*, 714–725.

Coifman, R.R., and Lafon, S. (2006). Diffusion maps. Appl. Comput. Harmon. Anal. *21*, 5–30.

Friedman, J., Hastie, T., and Tibshirani, R. (2010). Regularization Paths for Generalized Linear Models via Coordinate Descent. J. Stat. Softw. *33*, 1–22.

Kouno, T., de Hoon, M., Mar, J.C., Tomaru, Y., Kawano, M., Carninci, P., Suzuki, H., Hayashizaki, Y., and Shin, J.W. (2013). Temporal dynamics and transcriptional control using single-cell gene expression analysis. Genome Biol. *14*, R118.

Van Der Maaten, L., and Hinton, G. (2008). Visualizing Data using t-SNE. J. Mach. Learn. Res. *9*, 2579–2605.

Stephens, M.A. (1970). Use of the Kolmogorov-Smirnov, Cramer-Von Mises and Related Statistics Without Extensive Tables. J. R. Stat. Soc. Ser. B *32*, 115–122.
